# A virus‐derived microRNA targets immune response genes during SARS‐CoV‐2 infection

**DOI:** 10.15252/embr.202154341

**Published:** 2021-12-16

**Authors:** Meetali Singh, Maxime Chazal, Piergiuseppe Quarato, Loan Bourdon, Christophe Malabat, Thomas Vallet, Marco Vignuzzi, Sylvie van der Werf, Sylvie Behillil, Flora Donati, Nathalie Sauvonnet, Giulia Nigro, Maryline Bourgine, Nolwenn Jouvenet, Germano Cecere

**Affiliations:** ^1^ Department of Developmental and Stem Cell Biology Institut Pasteur CNRS UMR3738, Mechanisms of Epigenetic Inheritance Université de Paris Paris France; ^2^ Virus Sensing and Signaling Unit Institut Pasteur CNRS UMR3569 Université de Paris Paris France; ^3^ Department of Computational Biology Institut Pasteur, Bioinformatics and Biostatistics Hub Université de Paris Paris France; ^4^ Viral Populations and Pathogenesis Unit Institut Pasteur, CNRS UMR 3569 Université de Paris Paris France; ^5^ National Reference Center for Respiratory Viruses Molecular Genetics of RNA Viruses Institut Pasteur, CNRS UMR 3569 Université de Paris Paris France; ^6^ Intracellular Trafficking and Tissue Homeostasis Institut Pasteur Université de Paris Paris France; ^7^ Microenvironment and Immunity Unit Institut Pasteur, INSERM U1224 Université de Paris Paris France; ^8^ Virology Department Institut Pasteur, Institut Pasteur‐TheraVectys Joint Lab Université de Paris Paris France

**Keywords:** Argonaute, COVID‐19, interferon response, miRNA, SARS‐CoV‐2, Microbiology, Virology & Host Pathogen Interaction, RNA Biology

## Abstract

SARS‐CoV‐2 infection results in impaired interferon response in patients with severe COVID‐19. However, how SARS‐CoV‐2 interferes with host immune responses is incompletely understood. Here, we sequence small RNAs from SARS‐CoV‐2‐infected human cells and identify a microRNA (miRNA) derived from a recently evolved region of the viral genome. We show that the virus‐derived miRNA produces two miRNA isoforms in infected cells by the enzyme Dicer, which are loaded into Argonaute proteins. Moreover, the predominant miRNA isoform targets the 3′UTR of interferon‐stimulated genes and represses their expression in a miRNA‐like fashion. Finally, the two viral miRNA isoforms were detected in nasopharyngeal swabs from COVID‐19 patients. We propose that SARS‐CoV‐2 can potentially employ a virus‐derived miRNA to hijack the host miRNA machinery, which could help to evade the interferon‐mediated immune response.

## Graphical abstract

SARS‐CoV‐2 produces two miRNAs derived from a conserved stem‐loop structure of the ORF‐7a transcript in a Dicer‐dependent manner. These virus‐derived miRNAs interact with host Argonaute and can repress host innate immune response genes in cultured cells.

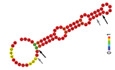

## Introduction

The infection by the severe acute respiratory syndrome‐related coronavirus 2 (SARS‐CoV‐2), which causes coronavirus disease 2019 (COVID‐19), is characterized by a wide range of symptoms, which in some cases lead to severe or critical disease outcomes, including pneumonia and acute respiratory failure (Huang *et al*, 2020; Salje *et al*, 2020). Several studies have highlighted the central role of interferons (IFNs) in the outcome of COVID‐19 disease (Acharya *et al*, 2020; Kim & Shin, 2021; Schultze & Aschenbrenner, 2021). The production of IFNs results in the activation of hundreds of interferon‐stimulated genes (ISGs), which are the effectors of the host innate antiviral response (Lazear *et al*, 2019). However, patients with severe and critical COVID‐19 disease manifestation show an impaired type I IFN response (Hadjadj *et al*, 2020). Moreover, several human cell lines, primary cells, and *in vivo* samples derived from COVID‐19 patients display a general impairment in activating ISGs upon SARS‐CoV‐2 infection (Bastard *et al*, 2020; Blanco‐Melo *et al*, 2020; Zhang *et al*, 2020a; Galani *et al*, 2021). In addition, different SARS‐CoV‐2 encoded proteins have now been shown to interfere with the interferon response (Lei *et al*, 2020; Miorin *et al*, 2020; Xia *et al*, 2020; Lin *et al*, 2021; Schroeder *et al*, 2021; Wu *et al*, 2021), implicating the importance on type I IFN response for counteracting SARS‐CoV‐2 infection.

Among the different mechanisms employed by viruses to interfere with host innate immune responses includes the use of small regulatory RNAs. Small RNAs, such as microRNAs (miRNAs), are fundamental regulators of host gene expression programs, including antiviral innate immunity genes (Girardi *et al*, 2018). They are produced by the host genome in regions that form stem–loop RNA structures (Kim *et al*, 2009), which are processed by the endoribonuclease enzyme Dicer resulting in small RNAs of approximately 22 nucleotides (nt) in length. The mature miRNA is then loaded by Argonaute proteins (AGOs), a part of the RNA silencing effector complex that regulates target transcripts by sequence complementarity (Bartel, 2018). Dicer also cleaves double‐stranded RNAs (dsRNAs) derived from RNA viruses’ genomes to inhibit viral replication and induce viral immunity through the production of small interfering RNAs (siRNAs) (Berkhout, 2018). Moreover, miRNAs can also be derived from viral genomes and processed by the host miRNA pathway (Mishra *et al*, 2020). The functions of virus‐derived miRNAs are still not fully understood. However, in some cases, the virus can employ miRNAs to evade the host immune response (Mishra *et al*, 2020). Given that some of the host enzymes involved in the biogenesis of miRNAs localize to the nucleus (Kim *et al*, 2009), all known viral miRNAs are derived by viruses replicating into the nucleus (Mishra *et al*, 2020). Nonetheless, cytoplasmic RNA viruses carrying artificial miRNA sequences can produce mature miRNAs (Rouha *et al*, 2010; Langlois *et al*, 2012; Shapiro *et al*, 2012). Whether miRNAs are derived from cytoplasmic RNA viruses remains controversial. Here, we have identified a *bona fide* SARS‐CoV2‐derived miRNA which can target and regulate the expression of host genes involved in interferon response.

## Results

**Figure EV1:**
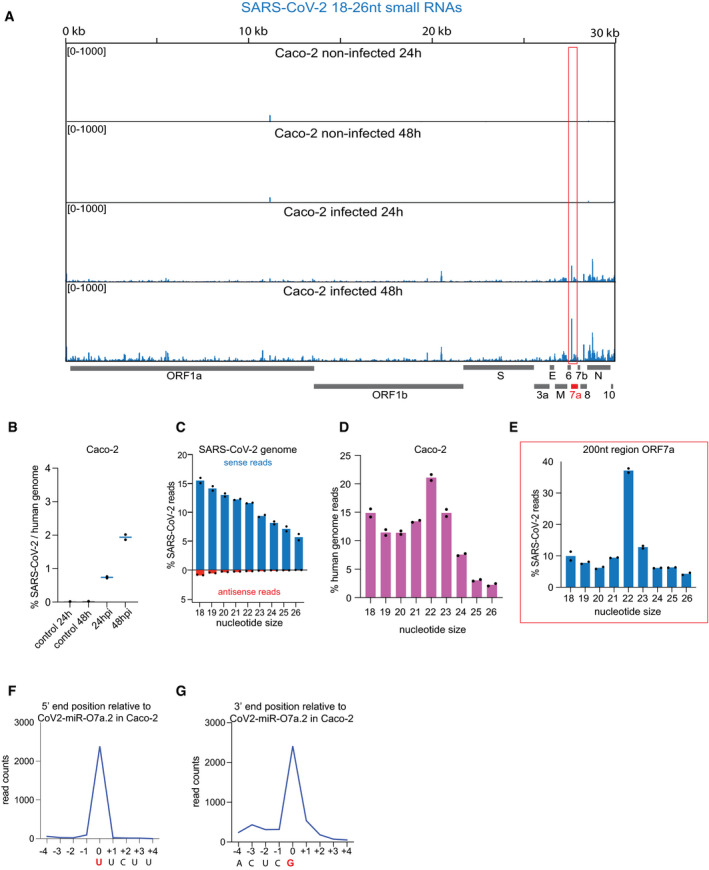
Identification of SARS‐CoV‐2 miR‐O7a in Caco‐2 human cells SARS‐CoV‐2 genomic view showing the distribution of normalized total small RNA reads (18–26 nt in length) from Caco‐2 cells at 24 and 48 hpi and non‐infected controls. The red box marks a distinct peak observed in ORF7a that has been further characterized *n* = 2.Percentage of total small RNA reads (18–26 nt) mapping on SARS‐CoV‐2 genome compared to the human genome from SARS‐CoV‐2 in Caco‐2 cells at 24 and 48 hpi and non‐infected controls. Line represents the average and individual dots represent data from two experiments.Percentage of the size distribution of SARS‐CoV‐2 total small RNA sense (blue) and antisense (red) reads from Caco‐2 cells at 48 hpi. Bars represent the average, and individual dots represent data from two experiments.The size distribution of total small RNA reads mapping on the human genome from Caco‐2 cells at 48 hpi shows a bias for 22 nt. Bars represent the average and individual dots represent data from two experiments.Percentage of the size distribution of small RNA reads from Caco‐2 cells at 48 hpi that map to the 200 nt region surrounding the distinct small RNA peak identified in ORF7a (red box in panel A). A bias for 22 nt typical of Dicer processed small RNAs is revealed. Bars represent the average, and individual dots represent data from two experiments.Distribution of 5′ end position relative to the most abundant small RNA derived from the ORF7a ranging from 26 to 18nt in Caco‐2 cells.Distribution of 3′ end position relative to the most abundant small RNA derived from the ORF7a ranging from 26 to 18nt in Caco‐2 cells. Source data are available online for this figure.

**Figure EV2:**
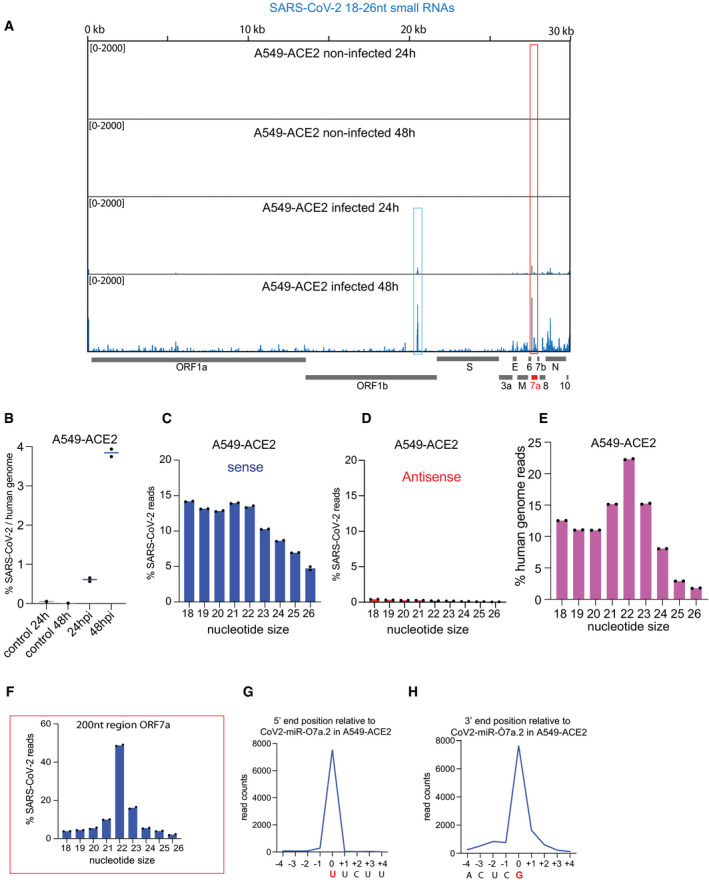
Identification of SARS‐CoV‐2 miR‐O7a in A549‐ACE2 human cells ASARS‐CoV‐2 genomic view showing the distribution of normalized total small RNA reads from infected A549‐ACE2 cells at 24 hpi and 48 hpi and non‐infected controls. The red box marks a distinct peak observed in ORF7a that has been further characterized. The blue box marks another peak derived from ORF1b, which is not abundant in Caco‐2 infected cells.BPercentage of total small RNA reads (18–26 nt) mapping on SARS‐Cov‐2 genome compared to the human genome from SARS‐CoV‐2 in A549‐ACE2 cells at 24 and 48 hpi and non‐infected controls. Line represents the average and individual dots represent data from two experiments.C, DThe size distribution of SARS‐CoV‐2 total small RNA sense (blue), (C) and antisense (red), (D) reads from A549‐ACE2 cells at 48 hpi. Bars represent the average, and individual dots represent data from two independent experiments.EThe size distribution of total small RNA reads mapping on the human genome from A549‐ACE2 cells at 48 hpi shows a bias for 22 nt. Bars represent the average, and individual dots represent data from two independent experiments.FThe size distribution of small RNA reads from A549‐ACE2 cells at 48 hpi that map to the 200 nt region surrounding the distinct small RNA peak identified in ORF7a (red box in panel E). A bias for 22 nt typical of Dicer processed small RNAs is revealed. Bars represent the average, and individual dots represent data from two independent experiments.GDistribution of 5′ end position relative to the most abundant small RNA derived from the ORF7a ranging from 26 to 18nt in A549‐ACE2 cells.HDistribution of 3′ end position relative to the most abundant small RNA derived from the ORF7a ranging from 26 to 18 nt in A549‐ACE2 cells. Source data are available online for this figure.

To analyze the repertoire of small RNAs produced upon SARS‐CoV‐2 infection with potential regulatory functions, we generated 5′ monophosphate‐dependent small RNA libraries from human colorectal adenocarcinoma cells (Caco‐2), an intestinal cellular model, and human pulmonary ACE2‐expressing A549 (A549‐ACE2) cells, both known to be highly susceptible to SARS‐CoV‐2 infection (Chu *et al*, 2020; Takayama, 2020). Specifically, we gel‐purified and sequenced small RNAs, ranging from 18 to 26 nucleotides (nt), at 24 and 48 h post‐infection (hpi) together with their respective non‐infected control cells. Our analysis revealed the presence of reads mapping to the SARS‐CoV‐2 genome in infected versus non‐infected cells (Dataset EV1). Furthermore, their amount increased throughout infection, indicating that these reads are produced during viral replication in both cellular models tested (Figs EV1A and B, and EV2A and B). Next, we analyzed their size distribution and directionality to verify whether these small RNAs are generated by Dicer from the viral dsRNA replication intermediates. We found a very small fraction of SARS‐CoV‐2 reads mapping in antisense orientation, and the majority of reads were not enriched for 22 nt reads (Figs EV1C and EV2C and D), the typical size of Dicer‐cleaved small RNAs (Bernstein *et al*, 2001). In contrast, the small RNAs mapped to the human genome, which included a large fraction of known human miRNAs (Dataset EV2), were enriched for small RNAs of 22 nt in length (Figs EV1D and EV2E). These results suggest that most SARS‐CoV‐2 reads do not represent canonical siRNAs generated by Dicer from dsRNA intermediates but instead result from degradation fragments of the viral RNA genome. Accordingly, most of the SARS‐CoV‐2 reads were distributed across the whole length of the viral genome (Figs EV1A and EV2A).

**Figure 1:**
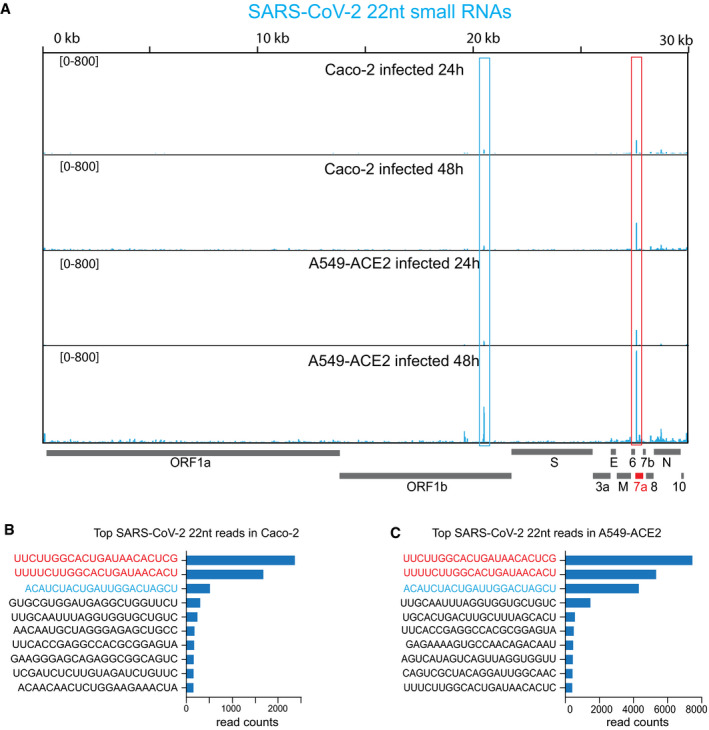
Identification of a virus‐derived miRNA during SARS‐CoV‐2 infection SARS‐CoV‐2 genomic view showing the distribution of normalized 22 nt small RNA reads from Caco‐2 and A549‐ACE2 cells at 24 and 48 hpi. The most abundant small RNAs are marked by the red and blue boxes. For all the experiments shown, *n* = 2.Total read counts for the ten most abundant 22 nt SARS‐CoV‐2 small RNAs in Caco‐2 cells at 48 hpi. The two most abundant small RNAs which differ by only 2 nt, marked in red, are derived from the ORF7a region marked by the red box in (A).Total read counts for the ten most abundant 22 nt SARS‐CoV‐2 small RNAs in A549‐ACE2 cells at 48 hpi. The two most abundant small RNAs derived from the ORF7a region marked by the red box in (A) are marked in red. The third abundant small RNA, marked in blue, derived from the ORF1b region marked by the blue box in (A). Source data are available online for this figure.

Intriguingly, we identified a well‐defined small RNA peak mapping to the beginning of the ORF7a, which increased in abundance upon viral replication (Figs EV1A and EV2A). The analysis of the size distribution of the reads across 200 nt surrounding the identified peak showed enrichment of 22 nt read lengths (Figs EV1E and EV2F). This result suggests that the small RNAs produced from this region do not result from the viral genome's degradation and could instead be virus‐derived miRNAs. To identify the precise sequences of these 22 nt small RNAs, we analyzed all the 22 nt reads that mapped to the SARS‐CoV‐2 genome (Fig 1A). Our analysis showed the presence of two predominant 22 nt small RNA sequences, which differ in only 2 nt and correspond to the identified peak at the beginning of the ORF7a in both Caco‐2 and A549‐ACE2 cells (Fig 1B and C). These results indicate that the two predominant 22 nt small RNAs derived from the ORF7a are produced in intestinal‐ and pulmonary‐derived human cell lines, representing tissues targeted by SARS‐CoV‐2 in humans (Wu *et al*, 2020). We further analyzed the size distribution of reads with the 5′ or the 3′ end ranging from 26 to 18nt for the most abundant small RNA derived from the ORF7a (Figs EV1F and G, and EV2G and H). This analysis revealed that the small RNA has precise 5′ and 3′ ends characteristic of a bonafide miRNA, suggesting that it is not a degradation product. In addition to the ORF7a, we identified another abundant 22 nt small RNA derived from ORF1b in A549‐ACE2 infected cells (Fig 1A and C). However, this small RNA is present at a much lower level in Caco‐2 cells (Fig 1A and B), and we thus decided to focus on the two small RNAs derived from the ORF7a.

**Figure 2:**
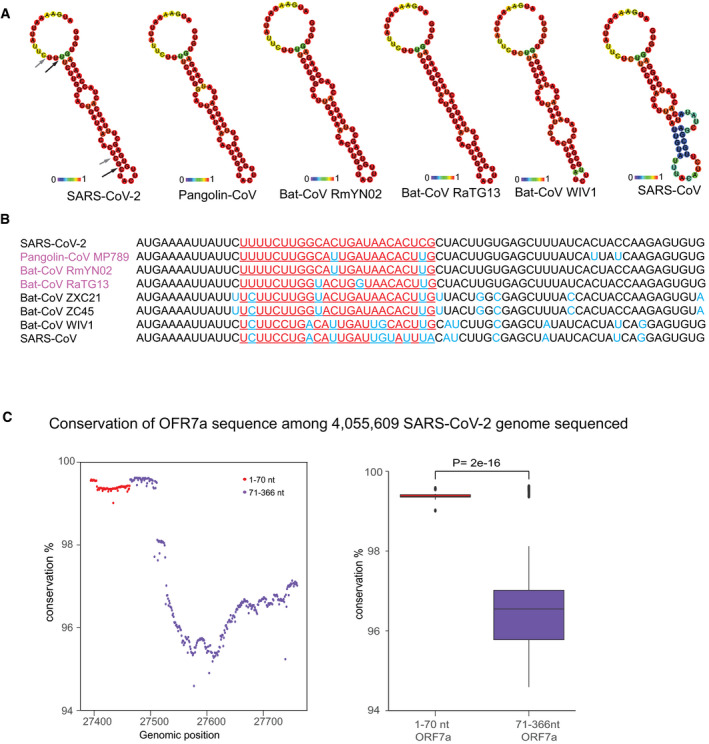
RNA secondary structure of SARS‐CoV‐2 miR‐O7a precursor and sequence conservation among different SARS coronaviruses and within SARS‐CoV‐2 variants Predicted RNA secondary structure for the CoV2‐miR‐O7a and flanking sequence using the first 70 nt of the open reading frame of the ORF7a. The arrows indicate the sites of the miRNAs possibly cleaved by Dicer. The stem–loop structure is not conserved in SARS‐CoV. The colors indicate the base pair probabilities.Conservation of the first 70 nt of the ORF7a sequence among different SARS coronaviruses. The underlined sequences are related to the position of the SARS‐CoV‐2 miR‐O7a. The conserved ribonucleotides of the CoV2‐miR‐O7a sequence are marked in red and in blue all the non‐conserved ribonucleotides across the 70nt sequence. The bat and pangolin coronaviruses closely related to SARS‐CoV‐2 are marked in purple.Percentage of conservation along the nucleotide positions in the ORF7a among 4,055,609 sequenced SARS‐CoV‐2 genomes. The first 70 nt are shown in red and show a higher percentage of conservation compared to the rest of the sequence of ORF7a. Boxplot shows the distribution of conservation percentage for each nucleotide either in the first 70 nt or 71–366 nt of ORF7a among 4,055,609 sequenced SARS‐CoV‐2 genomes. Box plots display median (line), first and third quartiles (box), and 5th /95th percentile value (whiskers). Each dot represents the outliers. Two‐tailed *P* values were calculated using Student’s *t*‐test.

**Figure EV3:**
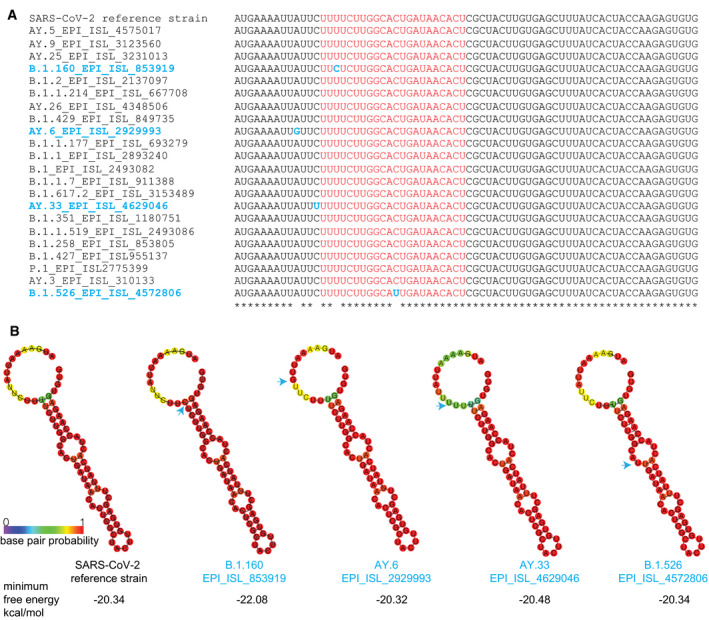
Conservation of first 70 nt and rest of the ORF‐7a among different variants of SARS‐CoV‐2 Conservation of the first 70 nt of the ORF7a sequence which forms the stem–loop structure among different SARS‐CoV‐2 variants. The underlined sequences are related to the position of the SARS‐CoV‐2 miR‐O7a. The conserved ribonucleotides of the CoV2‐miR‐O7a sequence are marked in red and in blue all the non‐conserved ribonucleotides across the 70nt sequence in different variants.Predicted RNA secondary structure for the CoV2‐miR‐O7a and flanking sequence using the first 70 nt of the open reading frame of the ORF7a for the SARS‐CoV‐2 reference strain and four variants with mutations in their sequences as highlighted in blue in (A). The arrows indicate the sites of mutation in the sequence. Minimal free energy for each of the stem–loop structures from different variants is shown. The colors indicate the base pair probabilities.

To verify that the two small RNAs can be viral‐derived miRNAs produced from a common stem–loop RNA precursor, we analyzed the first 70 nt of the ORF7a containing the two small RNAs. Indeed, our analysis predicts the formation of a stem–loop structure (Fig 2A), which could be recognized by Dicer to produce miRNAs (Lee *et al*, 2003). Thus, the two small RNAs generated from ORF7a are possibly two isoforms of the same virus‐derived miRNA, usually generated in mammals by imprecise Dicer cleavage of the same miRNA precursor (Chiang *et al*, 2010). Thus, we named these two small RNAs CoV2‐miR‐O7a.1 and CoV2‐miR‐O7a.2. Interestingly, the sequence producing the CoV2‐miR‐O7a is largely different in the SARS‐CoV genomes (Fig 2B), which does not generate the same stem–loop structure compatible with miRNA processing (Fig 2A). Instead, the bat RmYN02 and the pangolin MP789/2019 coronaviruses, which are closely related to SARS‐CoV‐2 (Zhou *et al*, 2020a, 2020b; Zhang *et al*, 2020b), showed a similar sequence and predicted stem–loop structure, unlike SARS‐CoV and other bat coronaviruses, suggesting the recent evolution of the CoV2‐miR‐O7a (Fig 2A and B). Next, we analyzed the degree of conservation within the ORF7a sequence among 4,055,609 genomic sequences of SARS‐CoV‐2 variants. We found that the first 70 nt encoding virus miRNAs are more conserved compared to the rest of the ORF7a among all the sequences of SARS‐CoV‐2 variants analyzed (Fig 2C and Appendix Fig S1). We further aligned the sequences of first 70 nucleotides that form the stem–loop structure from representative sequences of various SARS‐CoV‐2 variants to visualize mutations (Fig EV3A). Only four variants showed presence of single point mutations in the stem–loop region (Fig EV3A); however, none of the mutations had any impact on stem–loop structure or minimum free energy as can be seen by predicted structure (Fig EV3B). This result suggests that the beginning of the ORF7a is under selective pressure to maintain the sequence from which the two miRNAs are derived, which might be therefore biologically functional.

**Figure EV4:**
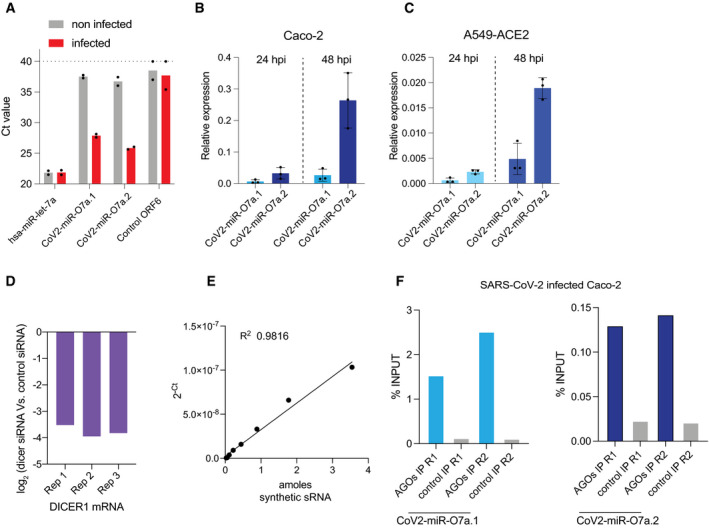
RT‐qPCR quantification of SARS‐CoV‐2 miR‐O7a in human and loading by AGOs ACt values for hsa‐miR‐let‐7a, CoV2‐miR‐O7a.1, CoV2‐miR‐O7a.2 and a 22 nt region from the ORF6 of the viral genome that produces a low level of small RNAs (Control ORF6) were determined by stem–loop RT‐qPCR performed in A549‐ACE2 cells. Bars represent the average, and individual dots represent data from two independent experiments.B, CExpression levels of CoV2‐miR‐O7a.1 and CoV2‐miR‐O7a.2 by stem–loop RT‐qPCR in Caco‐2 (B) and A549‐ACE2 cells (C) at 24 and 48 hpi. The mean and standard deviation of three experiments are shown. Relative expression to hsa‐miR‐let‐7a is shown.DLevels of DICER1 mRNA were analyzed by RT‐qPCR upon siRNA‐mediated DICER1 knockdown in A549‐ACE2 cells at 48 hpi compared with control siRNAs in the three biological replicates. Actin mRNA was used as internal control.EStandard curve with known amounts of synthetic small RNA measured by stem–loop RT‐qPCR for estimating copy number of host and viral miRNAs.FLoading of CoV2‐miR‐O7a.1 and COV2‐miR‐O7a.2 into AGOs as measured by stem–loop RT‐qPCR and analyzed as a percentage of input from the immunoprecipitates (IPs) of either pan‐AGO IP or control IgG IP from Caco‐2 cells at 48 hpi *n* = 2. Two independent replicates are shown. Source data are available online for this figure.

**Figure 3:**
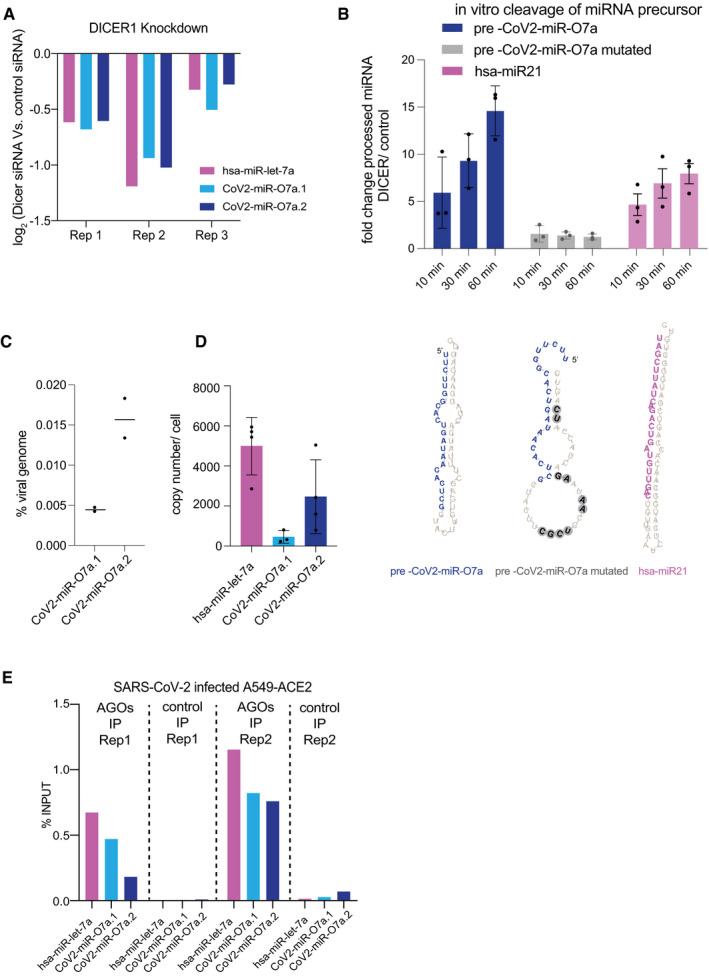
The SARS‐CoV‐2 miR‐O7a produces two isoforms processed by DICER and loaded onto AGOs Log_2_ fold changes of the levels of hsa‐miR‐let‐7a, CoV2‐miR‐O7a.1, and COV2‐miR‐O7a.2 in DICER1 knockdown SARS‐CoV‐2‐infected A549‐ACE2 cells compared to control cells analyzed by stem–loop RT‐qPCR. Results from three independent replicates are shown.
*In vitro* kinetics of DICER1‐mediated cleavage of pre‐CoV2‐miR‐O7a stem–loop precursor, pre‐CoV2‐miR‐O7a with mutations that prevent the formation of stem–loop structure and pre‐hsa‐miR21. Folded conformations for the three precursor molecules are shown. Fold change in the production of mature CoV2‐miR‐O7a.2 is shown in presence of DICER1 compared to control reaction (no DICER1) as measure by stem–loop RT‐qPCR. Bars and error bars represent the average and standard deviation from three independent experiments.Expression levels of the virus‐derived miRNAs as a percentage of the viral genome. Absolute quantification of virus‐derived miRNAs and viral genome from infected A549‐ACE2 cells was performed using two spike‐in (see methods). Line represents the average, and individual dots represent data from two independent experiments.Copy number per cell of hsa‐miR‐let‐7a, CoV2‐miR‐O7a.1, and CoV2‐miR‐O7a.2 in infected A549‐ACE2 cells quantified using a synthetic small RNA spike in standard curve by stem–loop RT‐qPCR. Levels of hsa‐miR‐let‐7a were normalized for the percentage of infected cells. Bars and error bars represent the average and standard deviation from at least three independent experiments.Loading of hsa‐miR‐let‐7a, CoV2‐miR‐O7a.1, and COV2‐miR‐O7a.2 into AGOs as measured by stem–loop RT‐qPCR and analyzed as a percentage of input from the immunoprecipitates (IPs) of either pan‐AGO IP or control IgG IP from infected A549‐ACE2 cells. Levels of miRNA were normalized for the percentage of infected cells. Source data are available online for this figure.

To test whether the human Dicer enzyme, DICER1, generates the CoV2‐miR‐O7a.1 and CoV2‐miR‐O7a.2, we used validated siRNAs to knock down DICER1 before SARS‐CoV‐2 infection in A549‐ACE2 cells. We first validated the production of the CoV2‐miR‐O7a.1 and CoV2‐miR‐O7a.2 by a stem–loop RT‐qPCR assay commonly used to detect mature miRNAs (Chen *et al*, 2005). As a result, we were able to specifically detect the CoV2‐miR‐O7a.1 and CoV2‐miR‐O7a.2 in A549‐ACE2‐infected cells (Fig EV4A). In contrast, no signal was obtained from a proximal region corresponding to the ORF6, which does not produce small RNAs (Fig EV4A). Moreover, we validated the sequencing results obtained in Caco‐2 and A549‐ACE2 cell lines showing that the CoV2‐miR‐O7a.2 is more abundant than the CoV2‐miR‐O7a.1 (Fig EV4B and C). Next, we performed an RT‐qPCR assay to detect the CoV2‐miR‐O7a.1 and CoV2‐miR‐O7a.2 in DICER1‐depleted cells. Our results showed that the depletion of DICER1 mRNA (Fig EV4D) was sufficient to reduce the levels of CoV2‐miR‐O7a.1 and CoV2‐miR‐O7a.2 (Fig 3A), and their reduction was similar to the decrease observed for the canonical dicer‐dependent human miR‐let‐7a (Fig 3A). These results suggest that CoV2‐miR‐O7a.1 and CoV2‐miR‐O7a.2 are two isoforms produced by the host enzyme Dicer. We validated this result by *in vitro* assay, where we analyzed cleavage of the stem–loop precursor substrate (pre‐CoV2‐miR‐O7a) by purified human DICER. Our results showed a time‐dependent increased production of mature CoV2‐miR‐O7a.2 when pre‐CoV2‐miR‐O7a was incubated with DICER1 compared to control conditions lacking DICER1 (Fig 3B). The kinetics of DICER1 cleavage of pre‐CoV2‐miR‐O7a was similar to that of host miRNA hsa‐miR21 (Fig 3B). We also introduced mutations in the pre‐CoV2‐miR‐O7a 3p arm without changing the CoV2‐miR‐O7a.2 sequence. This mutated sequence can no longer fold to a stem–loop conformation. DICER1 was not able to cleave this mutated pre‐CoV2‐miR‐O7a structure (Fig 3B), thus showing the specificity of DICER1 to recognize and cleave the pre‐CoV2‐miR‐O7a stem–loop structure to produce mature CoV2‐miR7a.2.

The processing of miRNAs directly from the viral RNA genome might be a strategy adopted by the host to reduce viral replication and could also explain why virus‐derived miRNAs are not prevalent in RNA viruses infected cells (Aguado & tenOever, 2018). However, we quantitatively measured the amount of CoV2‐miR‐O7a produced from the SARS‐CoV‐2 genome. We found that only 0.01% of the viral genome is used to produce the predominant isoform CoV2‐miR‐O7a, suggesting that it is unlikely that their production reduces viral genome copy numbers (Fig 3C). Next, we estimated the cell copy number of the viral miRNAs. We used a synthetic oligonucleotide to generate a standard curve to count the copy number of viral miRNA (Fig EV4E) (Chen *et al*, 2005) and found that more abundant CoV2‐miR‐O7a.2 isoform is present at ~2,465 copies per cell in infected A549‐ACE2 cells. This cell copy number is in a close range with one of the most abundant human miRNAs, the hsa‐miR‐let‐7a (~4,987 copies per cell) (Fig 3D, Dataset EV2). Given the abundance of CoV2‐miR‐O7a in infected human cells, we explored the possibility that the CoV2‐miR‐O7a hijacks the human AGOs to regulate host transcripts. To test this hypothesis, we performed RNA immunoprecipitation experiments in A549‐ACE2 cells using a pan‐AGO antibody that recognizes all four human AGOs (Nelson *et al*, 2007). Our results demonstrated the loading of CoV2‐miR‐O7a.1 and CoV2‐miR‐O7a.2 into human AGOs, with efficiency in a similar range to that of the human miR‐let‐7a (Fig 3E). We confirmed these results by RNA immunoprecipitation experiments performed in infected Caco‐2 cells (Fig EV4F). The *in vitro* processing of viral CoV2‐miR‐O7a by human Dicer, the requirement of Dicer for CoV2‐miR‐O7a accumulation in infected cells, and the loading onto human AGOs suggest that CoV2‐miR‐O7a might regulate human genes by hijacking the host miRNA machinery.

**Figure 4:**
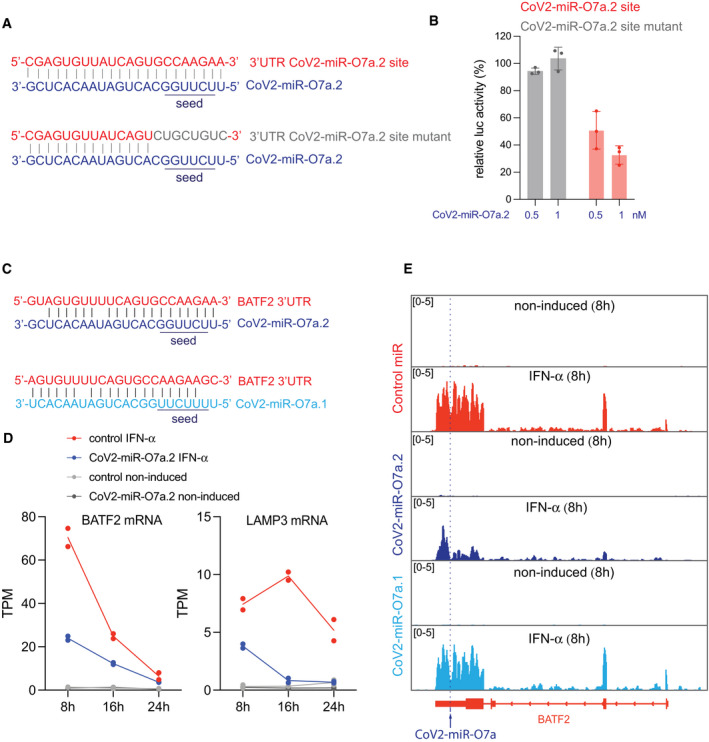
SARS‐CoV‐2 miR‐O7a.2 represses the activation of transcription factor BATF2 Base pairing of CoV2‐miR‐O7a.2 to complementary 3′UTR site or site with mutations in seed region cloned in 3′UTR of firefly luciferase mRNA in pmirGLO dual‐luciferase miRNA target expression vector. The seed region required for binding of miRNAs with its target is underlined.Relative firefly luciferase normalized to *Renilla* luciferase of pmirGLO dual‐luciferase miRNA target expression vector with either CoV2‐miR‐O7a.2 target site or CoV2‐miR‐O7a.2 site with mutations in seed region at 24 h on co‐transfection with CoV2‐miR‐O7a.2 or control miRNA mimics. Activity on transfecting control miRNA mimic was considered 100%. Bars and error bars represent the average and standard deviation from three independent experiments.Base pairing of CoV2‐miR‐O7a.2 to complementary 3′UTR site of BATF2 mRNA. The seed region required for binding of miRNAs with its target is underlined. One mismatch in the seed region of CoV2‐miR‐O7a.1 is observed.Kinetics of BATF2 and LAMP3 mRNA levels in non‐induced and interferon IFN‐α‐induced (for 8, 16, and 24 h) A549‐ACE2 cells transfected with either control or CoV2‐miR‐O7a.2 mimics. Normalized read abundances in transcript per million (TPM) are shown for two individual experiments (*n* = 2).Genomic view of the human BATF2 gene showing normalized RNA‐seq reads from non‐induced and IFN‐α‐induced (for 8 h) A549‐ACE2 cells transfected with either control, CoV2‐miR‐O7a.1, or CoV2‐miR‐O7a.2 mimics. The complementary site of CoV2‐miR‐O7a to BATF2 3′UTR is shown. Source data are available online for this figure.

**Figure EV5:**
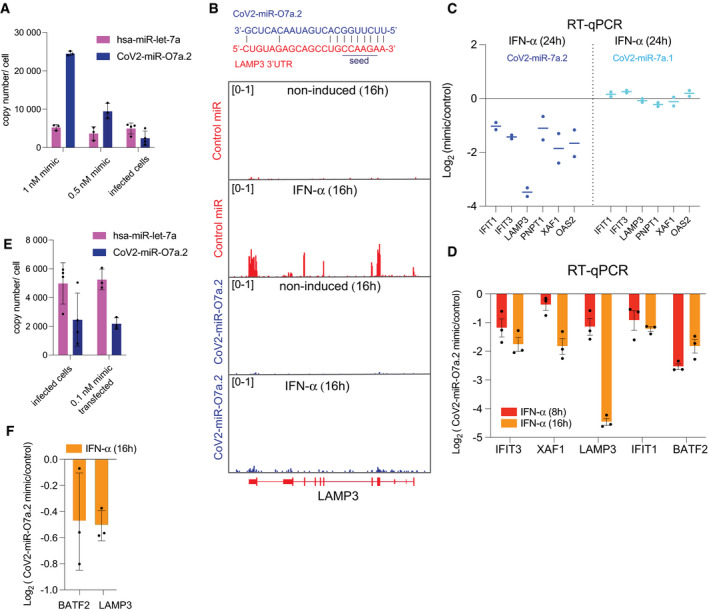
Regulation of ISGs by SARS‐CoV‐2 miR‐O7a across different time points of IFN‐α treatment Copy number per cell of CoV2‐miR‐O7a.2 transfected in A549‐ACE2 cells for luciferase reporter experiment (Fig 4B) and hsa‐let7a compared to SARS‐CoV‐2 infected cells. The mean and standard deviation of three experiments are shown. Data for virus‐infected cells are shown again in this plot from Fig 3D for ease of comparison.Genomic view of the human LAMP3 gene showing normalized RNA‐seq reads from non‐induced and IFN‐α‐induced (for 16 h) A549‐ACE2 cells transfected with CoV2‐miR‐O7a.2 or control mimics. The base pairing of CoV2‐miR‐O7a.2 to complementary 3′UTR site of LAMP3 is shown above and the seed region required for binding of miRNAs with the target is underlined.Log_2_ fold change of expression of selected ISGs measured by RT‐qPCR in IFN‐α ‐treated A549‐ACE2 cells transfected with CoV2‐miR‐O7a.2 or COV2‐miR‐O7a.1 compared to control mimic at 24 h upon IFN‐α treatment. Line represents the average, and individual dots represent data from two independent experiments.Log_2_ fold change of expression of selected ISGs measured by RT‐qPCR in IFN‐α ‐treated A549‐ACE2 cells transfected with CoV2‐miR‐O7a.2 mimic compared to control mimic at 8 and 16 h upon IFN‐α treatment. The mean and standard deviation of 3 experiments are shown.Copy number per cell of CoV2‐miR‐O7a.2 (0.1 nM) transfected in A549‐ACE2 cells for 24 h along with hsa‐let7a. Cells were then further induced by IFN‐α for 16 h. The mean and standard deviation of three experiments are shown. Data for virus‐infected cells are shown again in this plot from Fig 3D for ease of comparison.Log_2_ fold change of expression of CoV2‐miR‐O7a targets, BATF2 and LAMP3, measured by RT‐qPCR in IFN‐α ‐treated A549‐ACE2 cells transfected with CoV2‐miR‐O7a.2 (samples from Fig EV5E) compared to control mimic at 16 h upon IFN‐α treatment. The mean and standard deviation of three experiments are shown. Source data are available online for this figure.

Animal miRNAs target sites are located in the 3′UTRs of mRNAs and decrease their expression through translation inhibition and mRNA decay (Bartel, 2018). To test the ability of CoV2‐miR‐O7a to regulate mRNA translation or decay, we transfected the A549‐ACE2 cells with a plasmid expressing dual‐luciferase (firefly luciferase and *Renilla* luciferase) under control of moderate‐strength PGK promoter with either the CoV2‐miR‐O7a.2 site or Cov2‐miR‐O7a.2 site with a mutation in the seed region at the 3′UTR of firefly luciferase (Fig 4A) (Zeng & Cullen, 2003; Bartel, 2018). *Renilla* luciferase is used as a control reporter for normalization. However, we could not test the effect of CoV2‐miR‐O7a.2 on the expression of the luciferase reporter during SARS‐CoV2 infection as the infection leads to a global shut off of translation by a multipronged strategy (Finkel *et al*, 2021). To overcome this limitation, we co‐transfected these cells with two concentrations of 22 nt miRNA mimics corresponding to the sequence of more abundant isoform CoV2‐miR‐O7a.2 and control sequence not targeting the human genome. We then measured firefly luciferase activity and normalized it with *Renilla* luciferase, and observed an average 50% reduction for firefly luciferase activity at 0.5 nM CoV2‐miR‐O7a.2 and a further decrease at 1 nM (Fig 4B). CoV2‐miR‐O7a.2 failed to silence firefly luciferase with CoV2‐miR‐O7a.2 site with a mutation in the seed region (Fig 4B). We calculated the copy number per cell of transfected CoV2‐miR‐O7a.2 mimic at the two concentrations, and an average copy number of 9,496 per cell at 0.5 nM was found to be in the range of cellular miRNA hsa‐miR‐let‐7a (Fig EV5A) (Chen *et al*, 2005).

To further investigate whether the CoV2‐miR‐O7a targets human genes, we analyzed the human transcriptome for homology to the sequence of CoV2‐miR‐O7a.1 and CoV2‐miR‐O7a.2. We found a nearly perfect antisense complementarity for the entire CoV2‐miR‐O7a.2 sequence to the 3′ untranslated regions (3′UTR) of BATF2 with 100% complementarity in the seed region (Fig 4C), which is a transcription factor that plays a major role in innate immunity during viral infection (Tussiwand *et al*, 2012; Murphy *et al*, 2013). Importantly, BATF2 is an interferon‐stimulated gene (ISG), and, as such, its expression is suppressed in numerous human cells infected with SARS‐CoV‐2 (Blanco‐Melo *et al*, 2020), including A549‐ACE2 cells. We thus hypothesized that the SARS‐CoV‐2 miRNA could similarly inhibit the expression of BATF2 mRNA. To test this hypothesis, we transfected A549‐ACE2 cells with 22 nt miRNA mimics corresponding to the sequence of CoV2‐miR‐O7a.1, CoV2‐miR‐O7a.2, or a control sequence not targeting the human genome. Given that IFNs induce BATF2 mRNA expression, we performed a time‐course experiment to evaluate the level of expression of ISGs in A549‐ACE2 cells at 8, 16, and 24 h after IFN‐α induction compared to non‐induced controls (Figs 4D and 6A, and Dataset EV3). We found that BATF2 is highly induced by 8 h of IFN‐α treatment and its expression decays at 16 and 24 h (Figs 4D and 6A). We thus transfected the CoV2‐miR‐O7a.1, CoV2‐miR‐O7a.2, or control mimics in A549‐ACE2 cells and analyzed the level of BATF2 mRNAs after 8 h of IFN‐α treatment compared to non‐induced control. Our results showed a severe downregulation of BATF2 mRNAs upon 8 h of IFN‐α treatment in cells transfected with CoV2‐miR‐O7a.2, but not in cells transfected with the CoV2‐miR‐O7a.1 mimic (Fig 4E). Given that the repressive function of miRNAs is achieved through the base complementarity at their 5′ position, between the 2nd and the 7th nucleotides—the seed region—the imperfect complementarity of CoV2‐miR‐O7a.1 and BATF2 3′UTR in this region might explain the lack of repression despite the overall extensive complementarity (Fig 4C and E). These results suggest that CoV2‐miR‐O7a.2, which is the predominant CoV2‐miR‐O7a isoform, can potentially interfere with the expression of BATF2 mRNA during SARS‐CoV‐2 infection through a mechanism similar to the one used by the host miRNA pathway to regulate mRNA targets.

**Figure 5:**
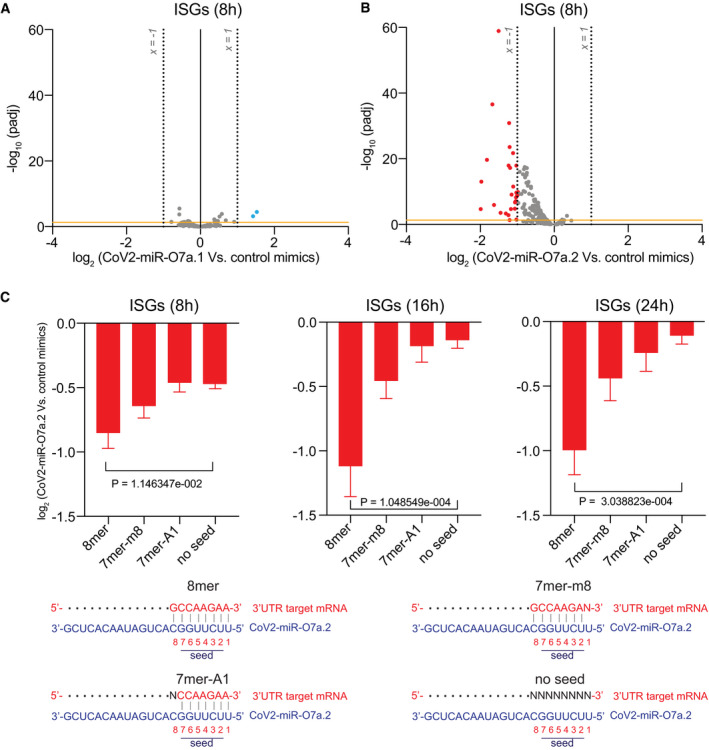
SARS‐CoV‐2 miR‐O7a.2 represses the activation of interferon‐stimulated genes with target sites in their 3′UTR A, BVolcano plots showing the log_2_ fold change and corresponding significance levels of ISGs upon 8 h of IFN‐α treatment in A549‐ACE2 cells transfected with CoV2‐miR‐O7a.1 (A) or CoV2‐miR‐O7a.2 (B) compared to control mimic. Significantly downregulated genes are marked in red and upregulated genes in blue. The orange horizontal line indicates two‐tailed *P* = 0.05. *n* = 2, dashed lines represent +1 and −1 log_2_ fold change.CLog_2_ fold change of ISGs at 8, 16, 24 h of IFN‐α treatment and categorized based on CoV2‐miR‐O7a.2 target sites 8mer, 7mer‐m8, 7mer‐A1, and no seed as shown in the schematic. The mean and standard error of the mean are shown. Number of ISGs with 8mer site *n* = 8, 7mer‐m8 *n* = 32, 7mer‐A1 *n* = 26, and no seed *n* = 92. The two‐tailed *P* values were calculated using the Mann–Whitney–Wilcoxon test. ISGs were calculated as all the upregulated genes (≥ 3‐fold; *P*adj < 0.05) in two replicates of IFN‐induced versus non‐induced conditions in all time points. Source data are available online for this figure.

**Figure 6:**
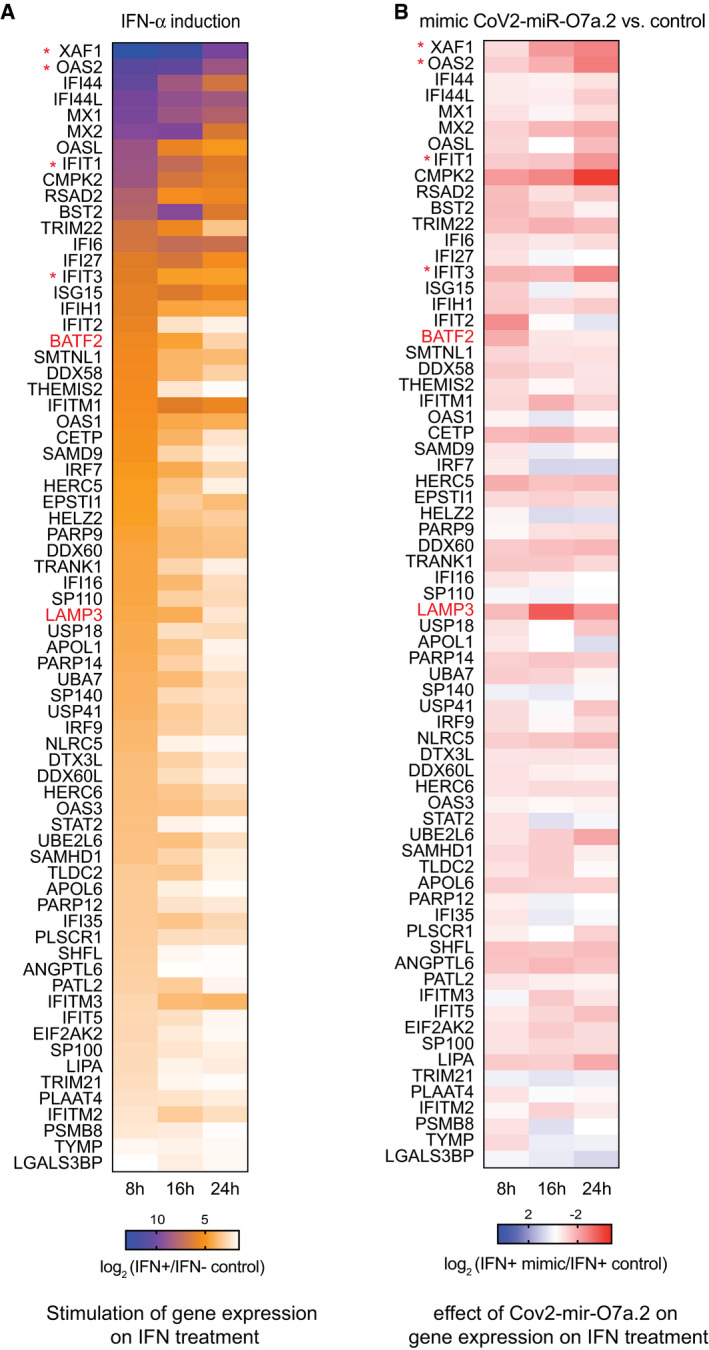
Dynamic expression of ISGs upon type I IFN‐α treatment at different time points in A549‐ACE2 cells and in the presence of CoV2‐miR‐O7a.2 mimic Heat map showing log_2_ fold change of expression of ISGs in A549‐ACE2 cells across 8, 16, and 24 h time points upon IFN‐α treatment compared to non‐treated controls. The common upregulated genes (≥ 3‐fold; *P*adj < 0.05) in IFN‐induced versus non‐induced conditions at all time points were categorized as ISGs.Heat map showing log_2_ fold change of expression of ISGs across 8, 16, and 24 h time points upon IFN‐α treatment in A549‐ACE2 cells transfected with CoV2‐miR‐O7a.2 mimic compared to control mimic. Data information: Genes marked in red and * indicate the ISGs detected by RT‐qPCR in Fig EV5C and D. Source data are available online for this figure.

Because miRNAs require only a few nucleotides to be perfectly complementary, also known as seed region, to the 3′UTR target, we identified putative targets of the viral miRNAs computationally, which included many ISGs with varying degrees of conservation of viral miRNA target sites (Dataset EV4). This observation suggests CoV2‐miR‐O7a may potentially regulate other ISGs, which have been shown to be important for the progression of COVID‐19 disease. We thus analyzed the changes in mRNAs of ISGs at 8 h of IFN‐α treatment compared to non‐induced controls in the presence of CoV2‐miR‐O7a.1, CoV2‐miR‐O7a.2 or the control sequence. Notably, we observed a global downregulation of ISGs in the presence of CoV2‐miR‐O7a.2, whereas the CoV2‐miR‐O7a.1 showed negligible effects on the expression of ISGs (Fig 5A and B). Even though the CoV2‐miR‐O7a.1 does not affect the expression of ISGs in the tested cell lines, we cannot rule out its effect on gene expression in other conditions. Our time‐course experiment using IFN‐α treatment also showed that some ISGs displayed a more stable expression than BATF2, which rapidly decay after induction by IFN‐α treatment (Figs 4D and 6A). Therefore, we tested whether the transfection of the CoV2‐miR‐O7a.2 mimic also shows inhibitory effects on ISGs at later time points of the IFN‐α treatment. Indeed, our experiment revealed increased global downregulation of ISGs at later time points upon IFN‐α stimulation in the presence of CoV2‐miR‐O7a.2 (Figs 5C and 6B). Moreover, the level of downregulation correlated with the degree of complementarity of the extended seed region—nucleotides 2‐8 and the presence of an adenosine across from the first nucleotide of the miRNAs (Fig 5C), similarly to what has been documented for canonical miRNA sites (Bartel, 2018). Indeed, the expression of one of the CoV2‐miR‐O7a.2 ISG targets with the most extended complementarity in the seed region, the dendritic cell lysosomal associated membrane glycoprotein LAMP3, was almost completely suppressed in the presence of CoV2‐miR‐O7a.2 at later time points (Figs 5D, 6B and EV5B). RT‐qPCR analyses on selected CoV2‐miR‐O7a.2 ISG targets at different time points of IFN‐α treatment confirmed our sequencing results (Fig EV5C and D). We also transfected the CoV2‐miR‐O7a.2 mimic at a lower concentration to mimic the copy number per cell as observed on the infection (~2,000 copies/cell) (Fig EV5E), and we found that at this concentration as well CoV2‐miR‐O7a.2 is able to downregulate both BATF2 and LAMP3 mRNA (Fig EV5F). Overall, these results suggest that the predominant isoform CoV2‐miR‐O7a.2 can potentially inhibit the expression of ISGs during SARS‐CoV2 infection.

**Figure 7:**
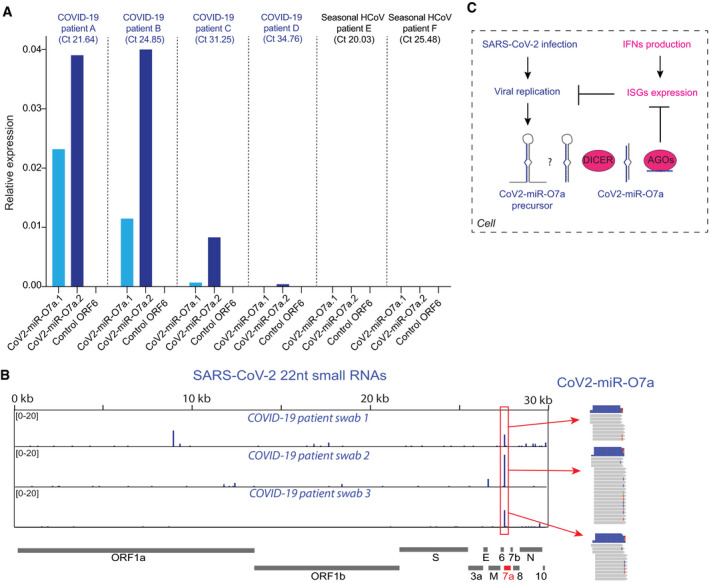
The two CoV2‐miR‐O7a isoforms are produced in COVID‐19 patients Expression levels of CoV2‐miR‐O7a.1, CoV2‐miR‐O7a.2, and a 22 nt region from the ORF6 of the viral genome that does not produce detectable levels of small RNAs (control ORF6) analyzed by stem–loop RT‐qPCR from nasopharyngeal swabs of patients tested positive for COVID‐19 or another seasonal human coronavirus (HCoV). Relative expression to hsa‐miR‐let‐7a is shown. Ct values in parenthesis refer to the Ct value for the detection of viral genome in patient swab samples.SARS‐CoV‐2 genomic view showing the distribution of normalized 22 nt small RNA reads from nasopharyngeal swabs of three patients tested positive for COVID‐19. The most abundant small RNAs are marked by the red boxes and correspond to the CoV2‐miR‐O7a.Model for the production and function of Cov2‐miR‐O7a in COVID‐19 patients. Source data are available online for this figure.

The detection of the CoV2‐miR‐O7a.1 and CoV2‐miR‐O7a.2 during infection of human cell lines does not provide evidence for their processing in more physiological conditions. We first evaluated the processing of the CoV2‐miR‐O7a.1 and CoV2‐miR‐O7a.2 using 2D human colon organoids, which are a relevant model to study SARS‐CoV‐2 biology and infection (Stanifer *et al*, 2020). Using this system, we detected by RT‐qPCR both CoV2‐miR‐O7a.1 and CoV2‐miR‐O7a.2 during SARS‐CoV‐2 infection at low and high multiplicity of infection (MOI) (Appendix Fig S2A and B). Moreover, as we observed in cell lines (Fig EV4B and C), the CoV2‐miR‐O7a.2 was the predominant isoform of the CoV2‐miR‐O7a in infected human intestinal 2D‐organoids (Appendix Fig S2A and B). To ensure that the observed processing of CoV2‐miR‐O7a does not result from a byproduct of *in vitro* cell culture, we tested the presence of CoV2‐miR‐O7a.1 and CoV2‐miR‐O7a.2 in COVID‐19 patients. We extracted small RNAs from nasopharyngeal swab samples collected from patients who tested positive for the presence of SARS‐CoV‐2 or from patients infected with seasonal human coronaviruses (HCoV). RT‐qPCR assays revealed the presence of the two CoV2‐miR‐O7a.1 and CoV2‐miR‐O7a.2 isoforms exclusively in COVID‐19 patients but not in HCoV‐infected patients (Fig 7A), while the human miR‐let‐7a was readily detected in all patients (Fig EV5C). Moreover, we failed to detect small RNA from the proximal ORF6 region, indicating that the amplification of the two isoforms of CoV2‐miR‐O7a is not the result of genomic viral RNA degradation in nasopharyngeal swab samples (Fig 7A and Appendix Fig S2C). In addition, the relative expression of the CoV2‐miR‐O7a.1 and CoV2‐miR‐O7a.2 among different COVID‐19 patients correlated with genomic viral RNA levels detected in the swab samples (Fig 7A), suggesting that the higher is the abundance of the SARS‐CoV‐2 genome, the higher is the processing of CoV2‐miR‐O7a.1 and CoV2‐miR‐O7a.2 by the human Dicer during viral replication in the upper respiratory tract. The relative levels of CoV2‐miR‐O7a.2 compared to CoV2‐miR‐O7a.1 confirmed that the CoV2‐miR‐O7a.2 is the predominant isoform of CoV2‐miR‐O7a in COVID‐19 patients (Fig 7A). Finally, we selected three nasopharyngeal swab samples collected from patients with high viral load to perform small RNA sequencing. Even though most of the reads were RNA degradation products, we were able to identify 22nt small RNA reads mapping to the CoV2‐miR‐O7a genomic regions (Fig 7B). This result confirms the production of CoV2‐miR‐O7a in human patients infected with SARS‐CoV‐2.

## Discussion

Multiple reports have shown computationally presence of SARS‐CoV‐2‐derived putative miRNAs; however, an experimental validation remained elusive (Demirci & Adan, 2020; Khan *et al*, 2020; Saini *et al*, 2020; Satyam *et al*, 2021). Our study identifies miRNAs derived from the cytoplasmic RNA virus SARS‐CoV‐2, which are processed by the host miRNA pathway and loaded by human AGOs. Several lines of evidence suggest that the viral small RNAs identified in the SARS‐CoV‐2 genome are *bona fide* miRNAs. First, the small RNA derived from the ORF7a of the SARS‐CoV‐2 genome shows precise 3′ and 5′ end, indicating that it is not a degradation product of the viral genome. Second, we show the presence of a stem–loop structure that is processed by DICER1‐mediated cleavage *in vitro*. Third, RNAi experiments to deplete human Dicer during SARS‐CoV‐2 infection also show reduced accumulation of the viral miRNA and human miRNAs. Fourth, human AGOs load the viral miRNA with similar efficiency as endogenous human miRNAs.

The precursor for CoV2‐miR‐O7a lacks a signature to suggest an involvement of DROSHA in the processing of the viral miRNA. Another independent preprint study presents evidence for production of CoV2‐miR‐O7a.2 upon SARS‐CoV‐2 infection using complementary approaches of sequencing and northern blot (preprint: Pawlica *et al*, 2021). They also show that the transfection of a CoV2‐miR‐O7a precursor in HEK‐293T DROSHA knockout cells generates the viral miRNA CoV2‐miR‐O7a.2 in a DROSHA‐independent manner (preprint: Pawlica *et al*, 2021). There have been well‐documented reports of DROSHA‐independent and DICER‐dependent miRNAs, including those that are processed from introns (mirtrons), snoRNA, and tRNA fragments that form a stem–loop structure recognized by Dicer (Okamura *et al*, 2007; Ruby *et al*, 2007; Babiarz *et al*, 2008; Treiber *et al*, 2019). For DROSHA‐independent miRNAs, flanking sequences for stem–loop precursors can be trimmed by cellular exonuclease including exosome complex (Flynt *et al*, 2010; Valen *et al*, 2011). Therefore, we propose that the CoV2‐miR‐O7a is processed in a drosha‐independent but dicer‐dependent manner. However, we cannot exclude the possibility that the CoV2‐miR‐O7a can also be produced in a dicer‐independent manner, similar to some AGO‐dependent miRNAs (Cheloufi *et al*, 2010; Cifuentes *et al*, 2010; Withers *et al*, 2019).

Our data further suggest that SARS‐CoV‐2 uses the viral miRNA to hijack the host miRNA machinery. The mature viral miRNAs are loaded by host Argonaute proteins and act like host miRNAs to potentially repress the expression of ISGs through sequence complementarity to sites located in their 3′UTR. We show with the aid of viral miRNA mimics that the level of suppression depends on the target site sequence conservation in the 3′UTR of ISGs. Thus, we propose that viral miRNA production is one of the mechanisms that may be used by the virus to potentially repress ISGs and evade the innate immune response (Fig 7C). Therefore, CoV2‐miR‐O7a.2 might contribute to the documented impaired activation of ISGs upon SARS‐CoV‐2 infection (Blanco‐Melo *et al*, 2020; Kim & Shin, 2021).

One of the hallmarks of severe COVID‐19 patients is the decreased expression of ISGs accompanied by low levels of type I IFN levels and high blood viral load (Hadjadj *et al*, 2020). Among the ISGs targeted by the CoV2‐miR‐O7a.2, two of the most regulated targets, BATF2, which plays a fundamental role during viral infections (Murphy *et al*, 2013), and LAMP3, which inhibits influenza virus replication (Zhou *et al*, 2011), are both required for dendritic cell function in adaptive immunity (Saint‐Vis *et al*, 1998; Tussiwand *et al*, 2012). Given that acute SARS‐CoV‐2 infection impairs dendritic cell response (Zhou *et al*, 2020c), we speculate that the suppression of key ISGs, including BATF2 and LAMP3, by the CoV2‐miR‐O7a might constitute one of the mechanisms responsible for the reduced dendritic cell response in patients with severe COVID‐19 disease, a hypothesis that needs further investigation. Furthermore, the sequence of the genomic region encoding CoV2‐miR‐O7a has been evolutionarily conserved in different variants of the SARS‐CoV‐2 and is less prone to mutations than adjoining genomic region, highlighting its potential biological relevance for viral infection. Although we cannot exclude that the viral miRNAs can potentially target other non ISGs genes, we did not observe a specific signature for that.

One limitation of this study is that we could not evaluate whether the presence and abundance of the CoV2‐miR‐O7a.2 correlate with disease progression or with impaired ISGs activation in patients with severe COVID‐19 disease outcomes. Thus, future studies will address the relevance of the CoV2‐miR‐O7a in the progression of the COVID‐19 disease. Furthermore, the recent evolution of the CoV2‐miR‐O7a sequence in the SARS‐CoV‐2 genome may facilitate the development of specific therapeutic approaches to potentially target and dampen the virulence of SARS‐CoV‐2 infection in COVID‐19 patients.

## Materials and Methods

### Ethical statement

Samples used in this study were anonymized and were collected as part of approved SARS‐CoV‐2 surveillance conducted by the National Reference Center for Respiratory Viruses at Institut Pasteur. The laboratory investigations were carried out in accordance with the General Data Protection Regulation (EU Regulation 2016/679 and Directive 95/46/EC) and the French data protection law (Law 78–17 on January 6, 1978, and Décret 2019–536 on May 29, 2019), which does not require a review by an ethics committee for the secondary use of samples collected for healthcare purposes. In such case, the secondary use for research is authorized if the individuals have been informed of such secondary use (article L.1211‐2 of the French Public Health Code).

### Human swab sample collection

For each suspected COVID‐19 case, respiratory samples from the upper respiratory tract (nasopharyngeal swabs) were sent to the NRC to perform SARS‐CoV‐2‐specific real‐time RT‐PCR.

### Cell culture

Human lung A549‐ACE2 cells, which have been modified to stably express ACE2 via lentiviral transduction, were generated in the laboratory of Pr. Olivier Schwartz (Institut Pasteur, Paris, France). Human colorectal adenocarcinoma Caco‐2 and African green monkey Vero E6 cells were purchased from ATCC. A549‐ACE2, Caco‐2, and Vero E6 cells were cultured in high‐glucose DMEM media (Gibco) supplemented with 10% fetal bovine serum (FBS; Sigma) and 1% penicillin–streptomycin (P/S; Gibco). Cells were maintained at 37°C in a humidified atmosphere with 5% CO_2_.

### Virus and infections

Experiments with SARS‐CoV‐2 isolates were performed in a BSL‐3 laboratory, following safety and security protocols approved by the risk prevention service of Institut Pasteur. The strain BetaCoV/France/IDF0372/2020 was supplied by the National Reference Centre for Respiratory Viruses hosted by Institut Pasteur (Paris, France) and headed by Pr. S. van der Werf. The human sample from which the strain was isolated has been provided by Dr. X. Lescure and Pr. Y. Yazdanpanah from the Bichat Hospital, Paris, France. Viral stocks were produced by amplification on Vero E6 cells, for 72 h in DMEM 2% FBS. The cleared supernatant was stored at −80°C and titrated on Vero E6 cells by using standard plaque assays to measure plaque‐forming units per ml (PFU/ml). A549‐ACE2 and Caco‐2 cells were infected at MOI of 3 and 0.3, respectively, in DMEM without FBS. After 2 h, DMEM with 5% FBS was added to the cells. 48 h post‐infection, cells were lysed using TRIzol LS reagent (Thermo Fischer Scientific) and RNA was extracted following manufacturer’s instructions or cells were lysed using FA buffer (50 mM HEPES pH 7.5, 1 mM EDTA, 1% Triton X‐100, 0.1% sodium deoxycholate, 150 mM NaCl, RNase inhibitor 40 U/ml, Halt™ Protease inhibitor cocktail 1×) for immunoprecipitation experiments. For measurement of miRNAs in the supernatant, RNA was isolated from the supernatant of infected cells using TRIzol LS reagent.

### Analysis of infected cells by flow cytometry

Flow cytometry analyses were performed for each experiment to evaluate the percentage of infected cells. Cells were fixed in 4% PFA for 20 min at 4°C and intracellular staining was performed in PBS, 1% BSA, 0.05% sodium azide, and 0.05% saponin. Cells were incubated with antibodies recognizing the spike protein of SARS‐CoV‐2 (anti‐S2 H2 162, a kind gift from Dr. Hugo Mouquet, Institut Pasteur, Paris, France) for 30 min at 4°C and then with secondary antibodies (anti‐human‐Alexa Fluor‐647) for 30 min at 4°C. Cells were acquired on an Attune NxT Flow Cytometer (Thermo Fisher) and data analyzed with FlowJo software.

### Luciferase reporter assay of miRNA activity

CoV2‐miR‐O7a.2 site or CoV2‐miR‐O7a.2 with mutated seed region (Fig 4A) was cloned at the 3′UTR of *luc‐2* in the pmirGLO Dual‐Luciferase miRNA Target expression vector (Promega E1330) according to manufacturer’s instruction. The sequence of oligonucleotides used for cloning is in Table EV1. 1 nM or 0.5 nM of CoV2‐miR‐O7a.2, or control mimics (Invitrogen) and 100 ng of pmirGLO Dual‐Luciferase miRNA Target expression vector with either CoV2‐miR‐O7a.2 site or CoV2‐miR‐O7a.2 with mutated seed region were transfected in A549‐ACE2 using Lipofectamine 3000 (Thermo Fischer Scientific) following the manufacturer’s instructions. 24 h post‐transfection, cells were lysed with passive lysis buffer (Promega) and firefly and *Renilla* luciferase activities were detected using Dual‐Glo Luciferase Assay System (Promega). Data are normalized by calculation of the ratio of luminescence from reporter firefly to internal control *Renilla* luciferase.

### Transfection of miRNA mimics and Interferon stimulation

1 nM of CoV2‐miR‐O7a.1, CoV2‐miR‐O7a.2, or control mimics (Invitrogen) were transfected in A549‐ACE2 or Caco‐2 cells using Lipofectamine RNAiMax (Thermo Fischer Scientific). 24 h post‐transfection, cells were treated with or without 100 U of human interferon alpha 2 (PBL Assay Science) for 8, 16, or 24 h. Cells were then lysed using TRIzol LS reagent (Thermo Fischer Scientific), and RNA was extracted following the manufacturer’s instructions or lysed in RIPA buffer (Thermo Fischer Scientific) for Western blot analysis. For mimic concentration of 0.1 nM A549‐ACE2 was transfected using Lipofectamine 3000 (Thermo Fischer Scientific) following the manufacturer’s instructions. 24 h post‐transfection, cells were treated with 100 U of human interferon alpha 2 (PBL Assay Science) for 16 h.

### siRNA‐mediated knockdown

A549‐ACE2 cells were transfected using Lipofectamine RNAiMax (Life Technologies) with 10 nM of control (#4390843, Ambion) or DICER1 siRNAs (#4390824, Ambion), following the manufacturer’s instructions. 48 h after transfection, cells were infected with SARS‐CoV‐2 for 24 h and then lysed using TRIzol LS reagent (Thermo Fischer Scientific).

### Infection of 2D colon organoids with SARS‐CoV‐2

Human tissues were a kind gift from Pr. Iradj Sobhani (Département de Gastroentérologie, Hôpital Henri Mondor, Créteil). They were collected from surgical resection in accordance with the recommendations of the hospital. Human colonic organoid cultures were generated from isolated crypts or from frozen tissues. They were maintained in culture for expansion prior to dissociation and plating on 0.4‐µm pore polyester membrane of Transwell^®^ inserts (Corning). Organoids were then recovered from the Matrigel using Cell recovery solution (Corning) and multiple steps of pipetting. After centrifugation, the organoid fragments were washed in cold DMEM and resuspended in the organoid medium. 2D‐organoids were seeded in chambers pre‐coated with 50 µg/ml human collagen IV (Millipore) for 2 h at 37°C. 700 µl of the medium was added to the bottom well of the chamber, and the cells were incubated at 37°, 5% CO_2_ for 2 days before changing the medium to the top compartment. The confluency of the monolayer was reached after 3–4 days of culture, and differentiation was induced for 4 days. Infection was performed as described above, by washing the cells after 2 h of virus incubation.

### Immunoprecipitation

A549‐ACE2 and Caco‐2 cells, infected and not, were lysed in FA buffer as described above. From the total lysate (~0.5 mg/ml), 10% lysate was saved as input and IP was performed using an anti‐pan‐AGO antibody (clone 2A8, MABE56 Sigma‐Aldrich) and an anti‐FLAG M2 antibody (F3165, Sigma‐Aldrich) was used for control IPs. The antibody was incubated with the lysates overnight at 4°C, followed by antibody capture by 40 µl Dynabeads™ Protein G (10003D, Invitrogen) for 3 h at 4°C on a rotor. Beads were then captured on a magnetic stand and washed four times with the FA buffer for 10 min at 4°C. After the final wash, the beads were captured on a magnetic stand and total RNA from input and the immunoprecipitate bound on beads was extracted using TRIzol™ Reagent as per manufacturer’s instruction.

### RNA extraction

For total RNA extraction, infected or non‐infected cells were directly lysed with TRIzol^TM^ LS (Invitrogen,) and total RNA was isolated according to the manufacturer’s instructions. For RNA‐seq or RT‐qPCR analysis, a maximum of 10 μg total RNAs was treated with 2 U Turbo DNase (Ambion) at 37°C for 30 min followed by acid phenol extraction and ethanol precipitation. An Agilent 2200 TapeStation System was used to evaluate the RIN indexes of all RNA preps, and only samples with RNA integrity number (RIN) > 8 were used for further investigations.

### RT‐qPCR

Reverse transcription for total RNA was performed using random hexamer primers according to the manufacturer’s instructions using M‐MLV reverse transcriptase (Invitrogen, Ref. 28025013). For RT of sRNAs, specific stem–loop RT primers were used (Table EV1) (Chen *et al*, 2005). Quantitative PCR (qPCR) was carried out using Applied Biosystems Power up SYBR Green PCR Master mix following the manufacturer’s instructions and using an Applied Biosystems QuantStudio 3 Real‐Time PCR System. Primers used for qPCR are listed in Table EV1. For host genes, GAPDH was used to normalize expression levels unless otherwise mentioned.

For absolute quantification of viral miRNAs in comparison for viral genome 1:1,000 dilution of ERCC RNA Spike‐In Mix (ERCC‐130 12 amoles) (Invitrogen, 4456740) and a custom sRNA oligo (GAGAGCAGUGGCUGGUUGAGAUUUAAU, 8 nmoles) were added to total RNA prior to RT. Known amounts of the spike‐ins were used for quantification of viral genome and miRNAs. Levels of hsa‐miR‐let‐7a were normalized to viral miRNAs as a ratio of total hsa‐miR‐let‐7a to the infection rate of infected cells. For copy number calculations, a standard curve was performed by RT‐qPCR for custom sRNA oligo (Chen *et al*, 2005). This standard curve was used to calculate the moles and further copies of each miRNA in a given sample. Copy number per cell was calculated by dividing total copies by the number of cells.

For immunoprecipitation experiments, levels of miRNAs were expressed as a percentage of input. For all other fold change comparisons, details are provided in figure legends.

### *In vitro* DICER‐1 assay

Stem–loop precursors for CoV2‐miR7a.2, mutant, and hsa‐miR21 were *in vitro*‐transcribed using oligonucleotides with T7 promoter (Table EV1). Oligonucleotides were annealed in oligo annealing buffer (Promega C838A) by denaturing at 94°C for 5 min and then cooling to 37°C for 15 min. *In vitro* transcription was performed according to manufacturer’s instructions using HiScribe™ T7 Quick High Yield RNA Synthesis Kit (NEB E2050S). *In vitro*‐transcribed RNA was purified after resolving on a 6% TBE‐Urea PAGE. RNA was extracted by crushing the gel and incubating in 0.3 M NaCl overnight at 25°C and further precipitated by 2‐propanol. Purified RNA was folded by incubating at 90°C for 5 min followed by 37°C for 15 min in 1× DICER assay buffer (50 mM NaCl, 3 mM MgCl_2_, 100 mM HEPES pH 7.5). DICER assay was performed using 640 fmols purified human DICER1 (Origene, TP319214) and 1 pmol of precursor RNA in 1× DICER assay buffer at 37°C for 10, 30, and 60 min (MacRae *et al*, 2007). Control assay without DICER1 proteins was performed. Samples were immediately purified using 3× volume 2‐propanol and 1.8× SPRI beads. Mature miRNAs were detected by stem–loop RT‐qPCR as described above. Ratio of mature miRNA in DICER1 assay and control assay was calculated.

### Small RNA‐seq

Total RNA (2–5 μg) was resolved on a 15% TBE‐urea gel (Invitrogen EC6885BOX). RNA of size between 17–25 nt was excised from the gel and extracted in 0.3 M NaCl overnight at 25°C. Size‐selected RNA was used to prepare libraries following previously described methodology (Barucci *et al*, 2020), which included the ligation of 3′ end and 5′ end adaptors each having four randomized nucleotides to minimize ligation biases. The randomized 8 nt was also used to remove possible PCR duplicates occurring in the PCR amplification step of the library preparation. We also exclusively ligated monophosphate small RNAs with a pre‐adenylated 3′ adaptor. Libraries were multiplexed and their quality was assessed on TapeStation (Agilent). They were quantified using the Qubit Fluorometer High Sensitivity dsDNA assay kit (Thermo Fisher Scientific, Q32851) and sequenced on a NextSeq‐500 Illumina platform using the NextSeq‐500/550 High Output v2 kit 75 cycles (FC‐404‐2005).

### Strand‐specific RNA‐seq library preparation

DNAse‐treated RNA with high RIN value was used to deplete ribosomal RNA using NEBNext^®^ rRNA Depletion Kit (Human/Mouse/Rat) (NEB #E6350) as per manufacturer’s instructions. Strand‐specific RNA libraries were prepared using at least 100 ng of rRNA depleted RNAs using NEBNext Ultra II Directional RNA Library Prep Kit for Illumina (E7760S) as per the manufacturer’s instructions.

### Data analysis

#### RNA‐seq

Multiplexed Illumina sequencing data were demultiplexed using Illumina bcl2fastq converter (version 2.20.0.422). Reads were aligned on the Homo sapiens genome (Build version GRCh38, NCBI) using Hisat2 (Kim *et al*, 2015) (version 2.2.1) with the default settings. After alignment, reads mapping to annotated protein‐coding genes were counted using featureCounts (version 2.0.1). Annotations were obtained from the Ensembl release 100. Counted reads for protein‐coding genes were used for differential expression analysis using the R/Bioconductor package DESeq2 (Love *et al*, 2014) (version 1.26.0).

#### Small RNA‐seq

Multiplexed Illumina sequencing data were demultiplexed using Illumina bcl2fastq converter (version 2.20.0.422). The 3′ adapter was trimmed from raw reads using Cutadapt (Martin, 2011) v.1.15 using the following parameter: ‐a TGGAATTCTCGGGTGCCAAGG ‐‐discard‐untrimmed. 5′ and 3′ end unique molecular identifiers (UMIs) were used to deduplicate the trimmed reads. Deduplication was performed by first sorting reads by sequence using the option ‐s in fastq‐sort (from fastq‐tools v.0.8; https://github.com/dcjones/fastq‐tools/tree/v0.8) and then using a custom Haskell program that retained the best quality reads at each position among reads of identical sequences. Then, 4‐nucleotide UMIs were trimmed at both ends using Cutadapt (options, ‐u 4 and ‐u −4). Finally, we selected only deduplicated reads ranging from 18 to 26 nucleotides using bioawk (https://github.com/lh3/bioawk). The selected 18–26‐nucleotide reads were aligned on the SARS‐CoV‐2 genome (assembly Jan.2020/NC_045512v2, UCSC) or on the Homo sapiens genome (Build version GRCh38, NCBI) using Bowtie2 (Li *et al*, 2009; Langmead & Salzberg, 2012) v.2.4.2 with the following parameters: ‐L 6 ‐i S,1,0.8 ‐N 0. Mapped reads were divided in sense and antisense reads in respect to the reference genome using samtools (Li *et al*, 2009) (version 1.3.1) while 22‐nucleotide reads were extracted from mapped reads using bioawk. The size distribution of all categories of mapped reads was calculated using bioawk. Reads mapping to CoV2‐microRNAs were counted using a custom script. First, a bed file with the coordinates of all the putative 22‐nucleotide RNAs encoded by the SARS‐CoV‐2 genome was created and used to extract their sequences using bedtools. Second, the occurrence of each putative 22‐nucleotide RNA among aligned reads was counted.

#### Generation of bigwig files

For RNA‐seq libraries, normalized bigwig files were generated from the mapping results using CPM as a normalization factor. This normalized coverage information was computed for 20 bp bins using the bamCoverage from deeptools (version 3.5.0).

For small RNA‐seq libraries, normalized bigwig files were generated from the mapping results using the sum of the total number of reads mapping on the SARS‐Cov‐2 genome or Homo sapiens genome as a normalization factor. This normalized coverage information was computed for 20 bp bins using the bamCoverage from deeptools (version 3.5.0).

#### Size distribution

Size distribution of mapped reads was computed using bioawk. To compute the size distribution around a specific region, reads mapping to a specific region of the genome were extracted using samtools and size distribution was computed using bioawk.

#### Identification of CoV2‐miR‐O7a.2 complementary sites on human 3′UTR

The sequences of the 3′UTR of human genes have been retrieved using the Ensembl BioMart (database Ensembl Genes 101—Human genes (GRCh38.p13)). Genes were divided into three different categories based on the presence in their 3′UTR of the miRNA complementary sites 8mer, 7mer‐m8, or 7mer‐A1 as described in (Bartel, 2018) (Dataset EV4).

#### RNA folding structure

RNA secondary structure of the CoV2‐miR‐O7a precursor region has been determined with the Vienna RNA Package (Hofacker, 2003) using the first 70 nt of the open reading frame of the ORF7a of different SARS coronaviruses.

#### Conservation analysis of viral genomic region encoding CoV2‐miR7a

A conservation study was performed on data obtained from GISAID (https://www.epicov.org/). A total of 4,055,609 sequences of SARS‐CoV2 were aligned, and the region of ORF7a (from position 27,394 to 27,759) was extracted for all sequences.

All sequences were compared to the original SARS‐CoV2 sequence (EPI_ISL_402124) and mutations were counted for all positions of the region. Conservation percentage was calculated as number of correct bases/total number of sequences. The same calculations were made on subsets of sequences corresponding to the major known variants. Variants were selected with atleast 14,000 sequenced samples. Main variants considered include with sequence counts mentioned in parenthesis for each: AY.25 (86,874), AY.26 (29,880), AY.3 (50,378), AY.33 (23,037), AY.4 (649,936), AY.5 (39,426), AY.6 (19,157), AY.9 (37,485), B.1.1.214 (18,063), B.1.1.519 (24,713), B.1.1.7 (1,091,214), B.1.1 (55,140), B.1.160 (29,236), B.1.177 (75,142), B.1.2 (107,599), B.1.258 (14,287), B.1.351 (32,074), B.1.427 (18,734), B.1.429 (41,141), B.1.526 (40,958), B.1.617.2 (516,787), B.1 (100,848), P.1 (73,400).

### Gene lists

Gene lists used in this study are shown in Dataset EV4, which also includes the log_2_ fold changes and padj used for all the analyses presented.

## Author contributions

GC identified and developed the core questions addressed in the project, including the identification of the viral miRNA. GC, MS, MC, and NJ designed the experiments and wrote the paper with the contribution of all the authors. MS performed all the biochemical, molecular biology and most of the sequencing experiments. PQ performed all the bioinformatic analyses. MC performed most of the cell culture and infection experiments. NJ supervised the cell culture and infection experiments. LB performed the RT‐qPCR experiments on human swab samples and on some human cell lines. CM performed the conservation analysis of the ORF7a among the SARS‐CoV2 sequenced genomes. TV and MV provided the first batch of infected cell lines. SvdW, SB, FD, provided the human swab samples and performed RNA extraction. NS and GN performed experiments on infected human 2D‐ organoids. MB contributed to some experiments. GC, MS, MC, and NJ analyzed the results.

## Conflict of interest

G.C. is an inventor on related CoV2‐miR‐O7a miRNAs European patent application EP 21305192.3 submitted in February 2021. All the other authors declare that they have no conflict of interest.

## Supporting information



Appendix

Expanded View Figures PDF

Table EV1

Dataset EV1

Dataset EV2

Dataset EV3

Dataset EV4

Source Data for Expanded View and Appendix

Source Data for Figure 1

Source Data for Figure 3

Source Data for Figure 4

Source Data for Figure 5

Source Data for Figure 6

Source Data for Figure 7

## Data Availability

All the sequencing data (RNA‐seq and sRNA‐seq) are available at the following accession numbers GSE162318 (https://www.ncbi.nlm.nih.gov/geo/query/acc.cgi?acc=GSE162318). All other data supporting the findings of this study are available from the corresponding author on request.
