# Supplementary material for: A virus‐derived microRNA targets immune response genes during SARS‐CoV‐2 infection
Source: EMBO Rep. 2021 Dec 16;23(2):e54341. doi: 10.15252/embr.202154341 (PMC8811647; doi:10.15252/embr.202154341)
Supplement: Supplementary file 1 — Appendix [file EMBR-23-e54341-s002.pdf]

Appendix for Paper "A virus-derived microRNA targets immune response genes during SARS-CoV-2 infection"

Table of Contents

Appendix Figure S1: Conservation of first 70 nt and rest of the ORF-7a among different variants of SARS-CoV-2. 2

Appendix Figure S2: Detection of CoV2-miR-O7a in 2D human colon organoids. 3

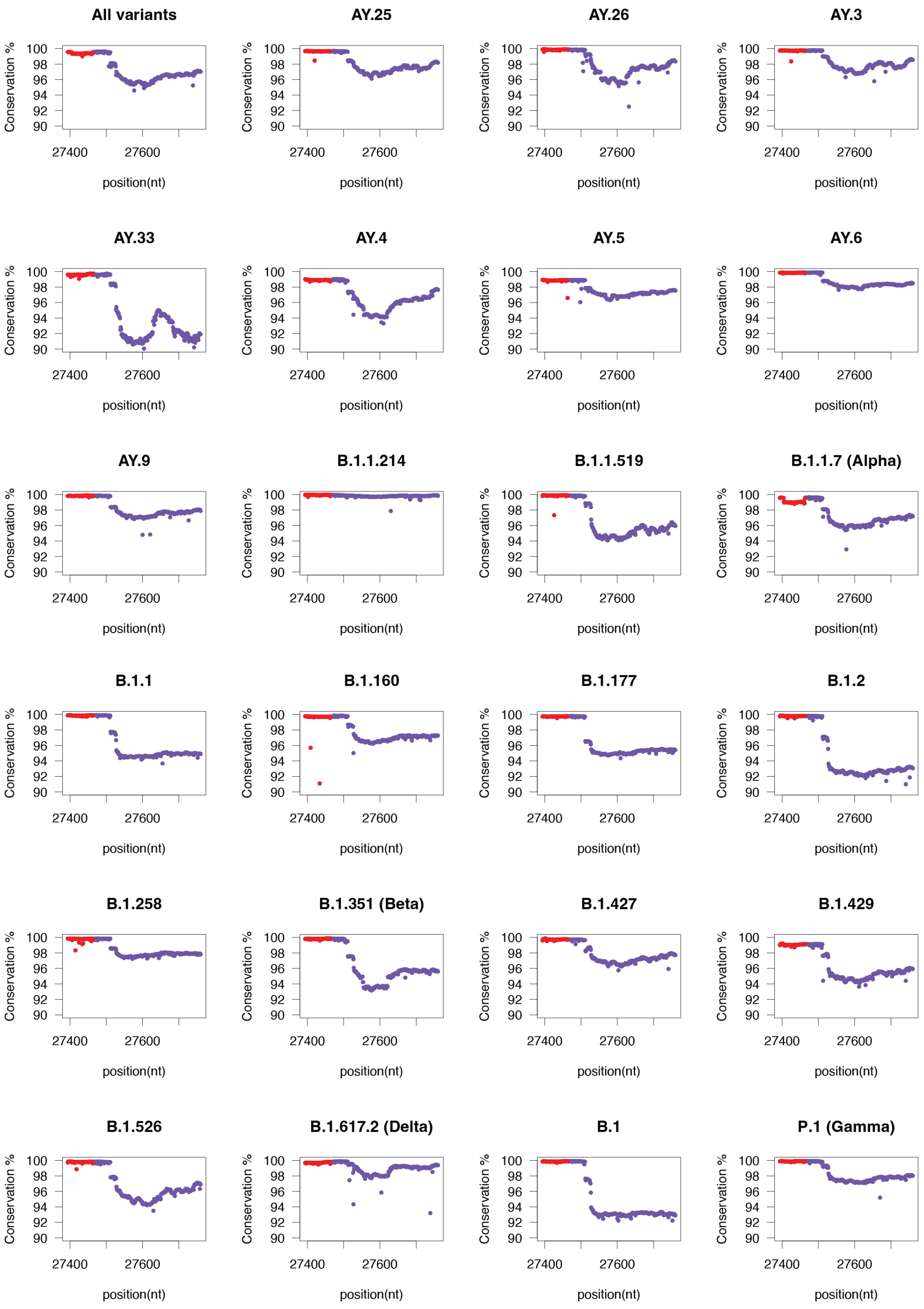

Appendix Figure S1: Conservation of first 70 nt and rest of the ORF-7a among different variants of SARS-CoV-2.

Percentage of conservation along the nucleotide positions in the ORF7a among different variants of SARS-CoV-2. The First 70 nt are shown in red and shows a higher percentage of conservation compared to the rest of the sequence of ORF-7a. The number of genome sequences analyzed for each variant can be found in materials and methods.

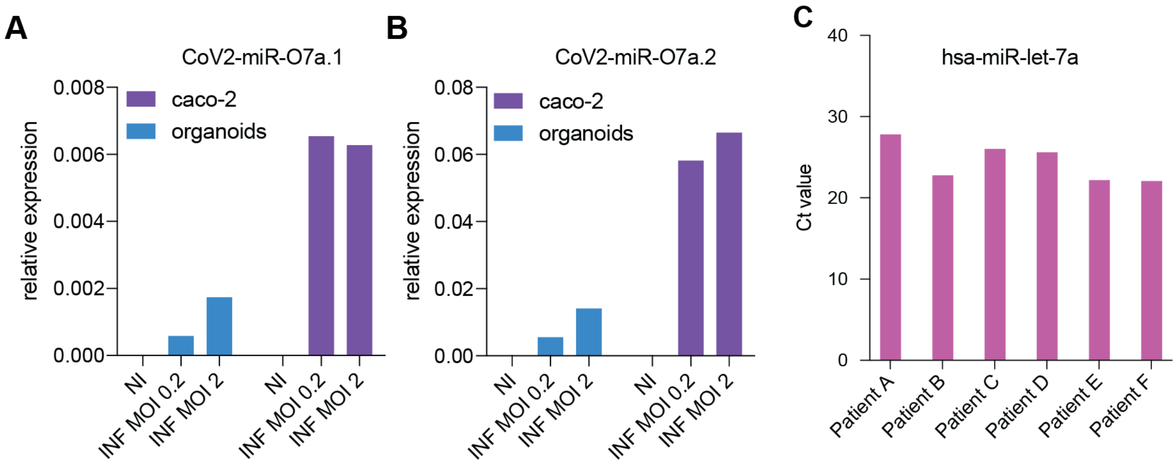

Appendix Figure S2: Detection of CoV2-miR-O7a in 2D human colon organoids.

(A-B) Detection of CoV2-miR-O7a.1 (A) and CoV2-miR-O7a.2 (B) by stem-loop RT-qPCR in no-infected (NI) and SARS-CoV-2-infected (INF) 2D human colon organoids and Caco-2 cells at an MOI of 0.2 and 2. Relative expression to hsa-miR-let-7a is shown.

(C) Ct values for hsa-miR-let-7a measured by stem-loop RT-qPCR from nasopharyngeal swabs of patients tested positive for COVID-19 or another seasonal HCoV (as in Fig. 7A).
