# Supplementary material for: A virus‐derived microRNA targets immune response genes during SARS‐CoV‐2 infection
Source: EMBO Rep. 2021 Dec 16;23(2):e54341. doi: 10.15252/embr.202154341 (PMC8811647; doi:10.15252/embr.202154341)
Supplement: Supplementary file 2 — Expanded View Figures PDF [file EMBR-23-e54341-s014.pdf]

Expanded View Figures

Figure EV1. Identification of SARS-CoV-2 miR-O7a in Caco-2 human cells.

A SARS-CoV-2 genomic view showing the distribution of normalized total small RNA reads (18–26 nt in length) from Caco-2 cells at 24 and 48 hpi and non-infected controls. The red box marks a distinct peak observed in ORF7a that has been further characterized $n = 2$.

B Percentage of total small RNA reads (18–26 nt) mapping on SARS-CoV-2 genome compared to the human genome from SARS-CoV-2 in Caco-2 cells at 24 and 48 hpi and non-infected controls. Line represents the average and individual dots represent data from two experiments.

C Percentage of the size distribution of SARS-CoV-2 total small RNA sense (blue) and antisense (red) reads from Caco-2 cells at 48 hpi. Bars represent the average, and individual dots represent data from two experiments.

D The size distribution of total small RNA reads mapping on the human genome from Caco-2 cells at 48 hpi shows a bias for 22 nt. Bars represent the average and individual dots represent data from two experiments.

E Percentage of the size distribution of small RNA reads from Caco-2 cells at 48 hpi that map to the 200 nt region surrounding the distinct small RNA peak identified in ORF7a (red box in panel A). A bias for 22 nt typical of Dicer processed small RNAs is revealed. Bars represent the average, and individual dots represent data from two experiments.

F Distribution of 5′ end position relative to the most abundant small RNA derived from the ORF7a ranging from 26 to 18nt in Caco-2 cells.

G Distribution of 3′ end position relative to the most abundant small RNA derived from the ORF7a ranging from 26 to 18nt in Caco-2 cells.

Source data are available online for this figure.

 © 2021 The Authors

18 of 17 EMBO reports 23: e54341 | 2022 © 2021 The Authors

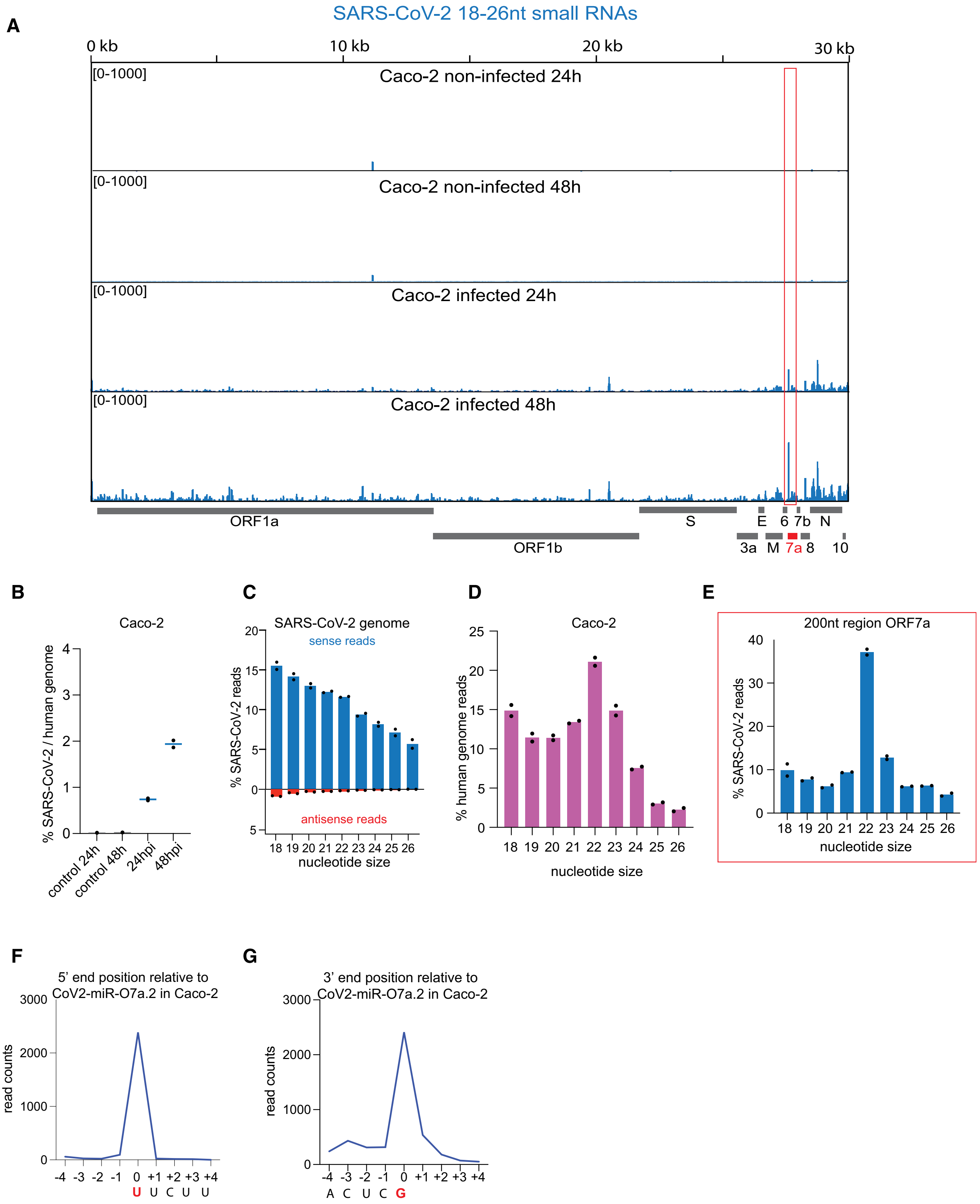
A
SARS-CoV-2 18-26nt small RNAs
0 kb
10 kb
20 kb
30 kb
[0-1000] Caco-2 non-infected 24h
[0-1000] Caco-2 non-infected 48h
[0-1000] Caco-2 infected 24h
[0-1000] Caco-2 infected 48h
ORF1a
ORF1b
S
E 6 7b N
3a M 7a 8 10
B
Caco-2
% SARS-CoV-2 / human genome
control 24h
control 48h
24hpi
48hpi
C
SARS-CoV-2 genome
sense reads
antisense reads
% SARS-CoV-2 reads
nucleotide size
D
Caco-2
% human genome reads
nucleotide size
E
200nt region ORF7a
% SARS-CoV-2 reads
nucleotide size
F
5' end position relative to CoV2-miR-O7a.2 in Caco-2
read counts
-4 -3 -2 -1 0 +1 +2 +3 +4
U U C U U
G
3' end position relative to CoV2-miR-O7a.2 in Caco-2
read counts
-4 -3 -2 -1 0 +1 +2 +3 +4
A C U C G

Figure EV1.

Figure EV2. Identification of SARS-CoV-2 miR-O7a in A549-ACE2 human cells.

A SARS-CoV-2 genomic view showing the distribution of normalized total small RNA reads from infected A549-ACE2 cells at 24 hpi and 48 hpi and non-infected controls. The red box marks a distinct peak observed in ORF7a that has been further characterized. The blue box marks another peak derived from ORF1b, which is not abundant in Caco-2 infected cells.

B Percentage of total small RNA reads (18–26 nt) mapping on SARS-Cov-2 genome compared to the human genome from SARS-CoV-2 in A549-ACE2 cells at 24 and 48 hpi and non-infected controls. Line represents the average and individual dots represent data from two experiments.

C, D The size distribution of SARS-CoV-2 total small RNA sense (blue), (C) and antisense (red), (D) reads from A549-ACE2 cells at 48 hpi. Bars represent the average, and individual dots represent data from two independent experiments.

E The size distribution of total small RNA reads mapping on the human genome from A549-ACE2 cells at 48 hpi shows a bias for 22 nt. Bars represent the average, and individual dots represent data from two independent experiments.

F The size distribution of small RNA reads from A549-ACE2 cells at 48 hpi that map to the 200 nt region surrounding the distinct small RNA peak identified in ORF7a (red box in panel E). A bias for 22 nt typical of Dicer processed small RNAs is revealed. Bars represent the average, and individual dots represent data from two independent experiments.

G Distribution of 5′ end position relative to the most abundant small RNA derived from the ORF7a ranging from 26 to 18nt in A549-ACE2 cells.

H Distribution of 3′ end position relative to the most abundant small RNA derived from the ORF7a ranging from 26 to 18 nt in A549-ACE2 cells.

Source data are available online for this figure.

 © 2021 The Authors

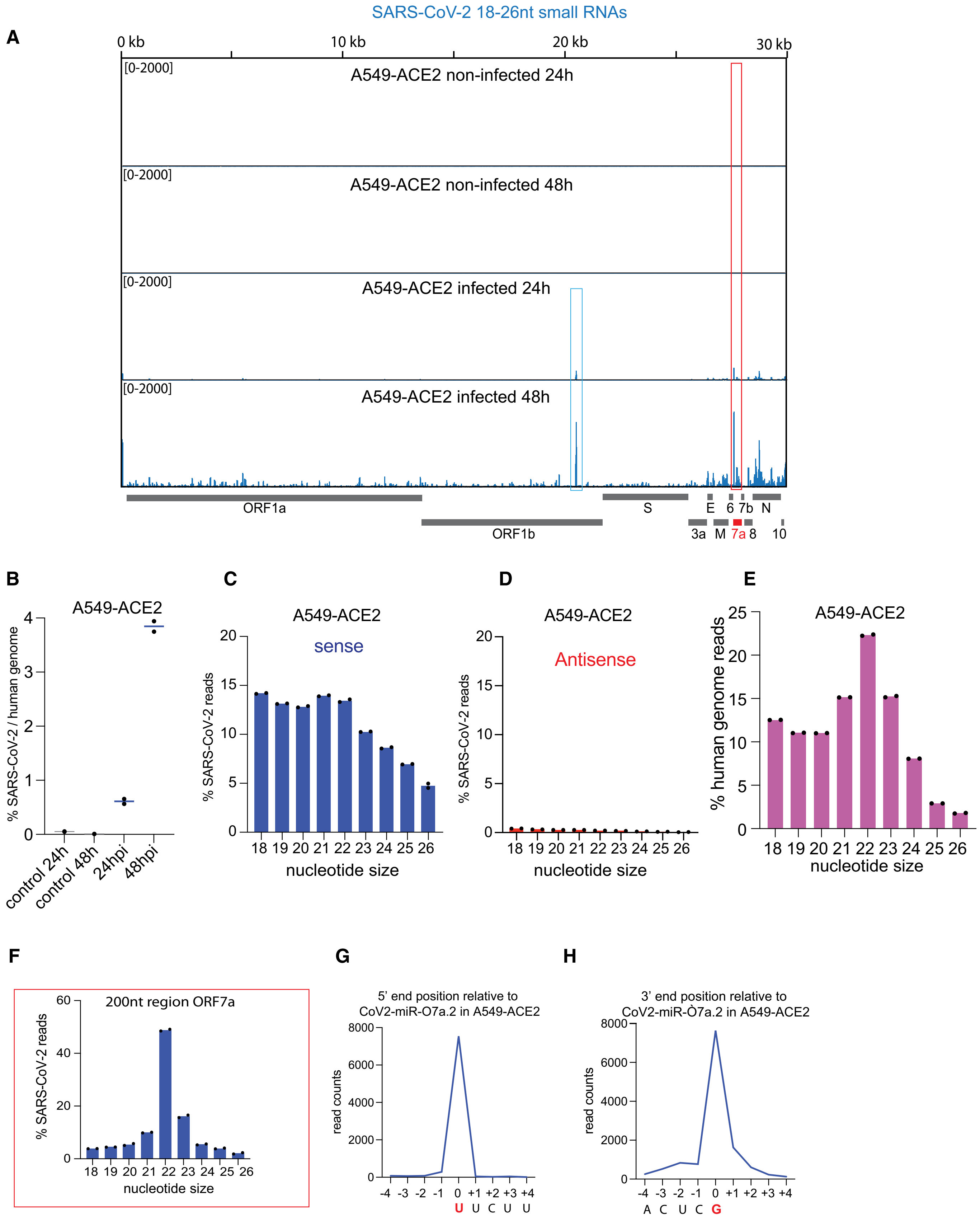
SARS-CoV-2 18-26nt small RNAs
A
0 kb
10 kb
20 kb
30 kb
[0-2000]
A549-ACE2 non-infected 24h
A549-ACE2 non-infected 48h
A549-ACE2 infected 24h
A549-ACE2 infected 48h
ORF1a
ORF1b
S
E 6 7b N
3a M 7a 8 10
B
A549-ACE2
% SARS-CoV-2 / human genome
control 24h
control 48h
24hpi
48hpi
C
A549-ACE2
sense
% SARS-CoV-2 reads
nucleotide size
D
A549-ACE2
Antisense
% SARS-CoV-2 reads
nucleotide size
E
A549-ACE2
% human genome reads
nucleotide size
F
200nt region ORF7a
% SARS-CoV-2 reads
nucleotide size
G
5' end position relative to CoV2-miR-O7a.2 in A549-ACE2
read counts
U U C U U
H
3' end position relative to CoV2-miR-Ò7a.2 in A549-ACE2
read counts
A C U C G

Figure EV2.

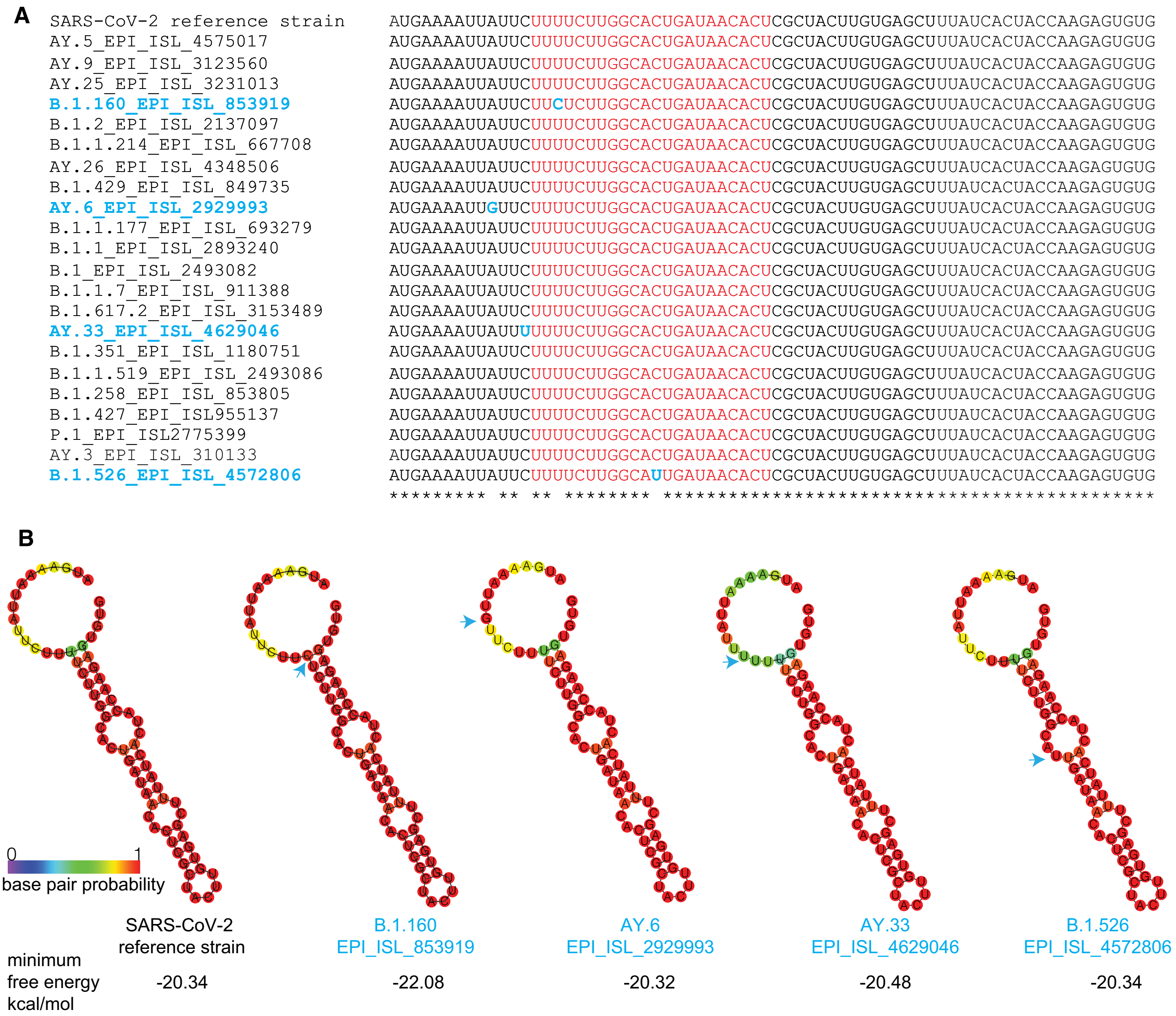

Figure EV3. Conservation of first 70 nt and rest of the ORF-7a among different variants of SARS-CoV-2.

A Conservation of the first 70 nt of the ORF7a sequence which forms the stem–loop structure among different SARS-CoV-2 variants. The underlined sequences are related to the position of the SARS-CoV-2 miR-O7a. The conserved ribonucleotides of the CoV2-miR-O7a sequence are marked in red and in blue all the non-conserved ribonucleotides across the 70nt sequence in different variants.

B Predicted RNA secondary structure for the CoV2-miR-O7a and flanking sequence using the first 70 nt of the open reading frame of the ORF7a for the SARS-CoV-2 reference strain and four variants with mutations in their sequences as highlighted in blue in (A). The arrows indicate the sites of mutation in the sequence. Minimal free energy for each of the stem–loop structures from different variants is shown. The colors indicate the base pair probabilities.

 © 2021 The Authors

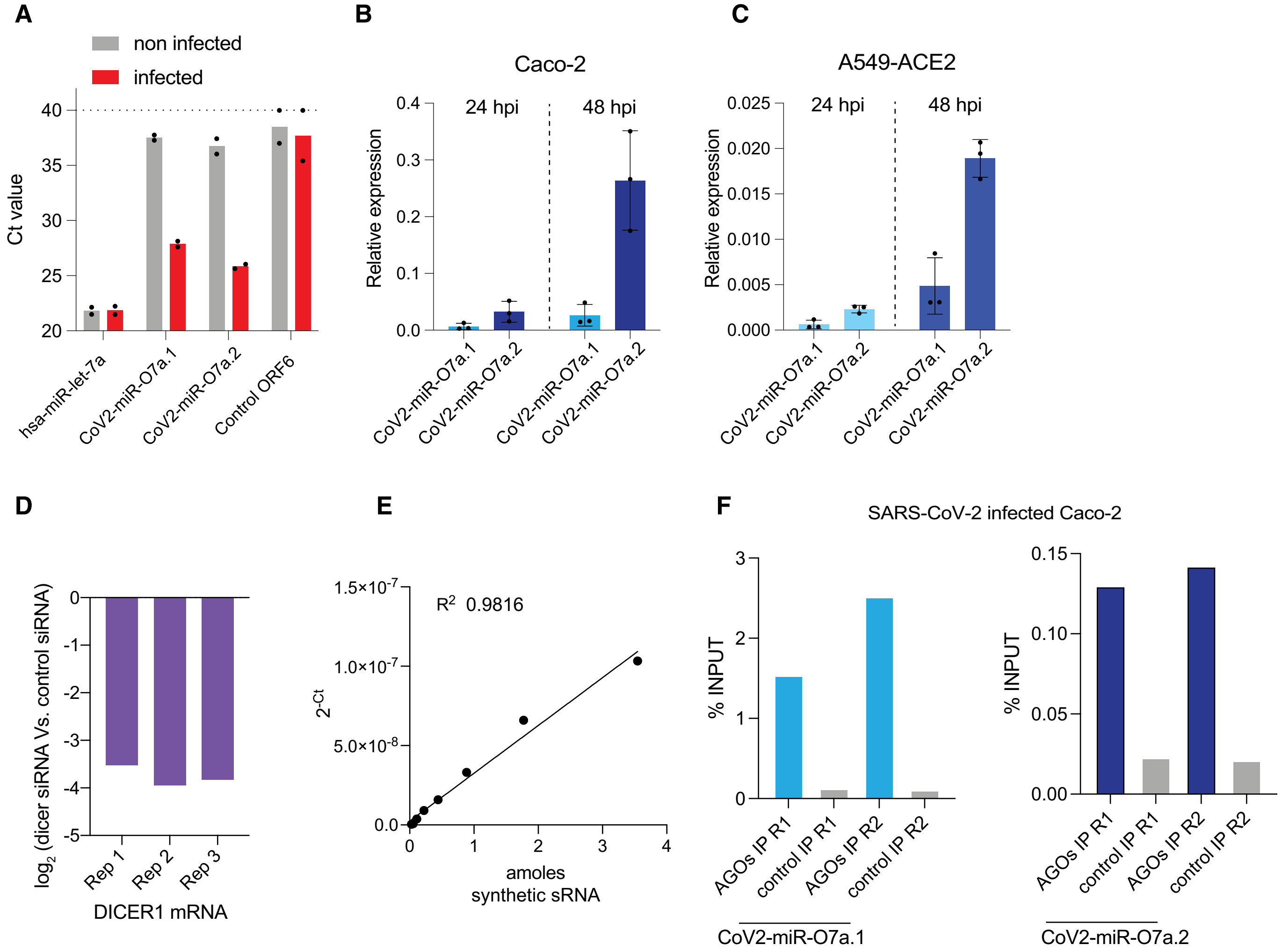

Figure EV4. RT-qPCR quantification of SARS-CoV-2 miR-O7a in human and loading by AGOs.

A Ct values for hsa-miR-let-7a, CoV2-miR-O7a.1, CoV2-miR-O7a.2 and a 22 nt region from the ORF6 of the viral genome that produces a low level of small RNAs (Control ORF6) were determined by stem–loop RT-qPCR performed in A549-ACE2 cells. Bars represent the average, and individual dots represent data from two independent experiments.

B, C Expression levels of CoV2-miR-O7a.1 and CoV2-miR-O7a.2 by stem–loop RT-qPCR in Caco-2 (B) and A549-ACE2 cells (C) at 24 and 48 hpi. The mean and standard deviation of three experiments are shown. Relative expression to hsa-miR-let-7a is shown.

D Levels of DICER1 mRNA were analyzed by RT-qPCR upon siRNA-mediated DICER1 knockdown in A549-ACE2 cells at 48 hpi compared with control siRNAs in the three biological replicates. Actin mRNA was used as internal control.

E Standard curve with known amounts of synthetic small RNA measured by stem–loop RT-qPCR for estimating copy number of host and viral miRNAs.

F Loading of CoV2-miR-O7a.1 and COV2-miR-O7a.2 into AGOs as measured by stem–loop RT-qPCR and analyzed as a percentage of input from the immunoprecipitates (IPs) of either pan-AGO IP or control IgG IP from Caco-2 cells at 48 hpi $n = 2$. Two independent replicates are shown.

Source data are available online for this figure.

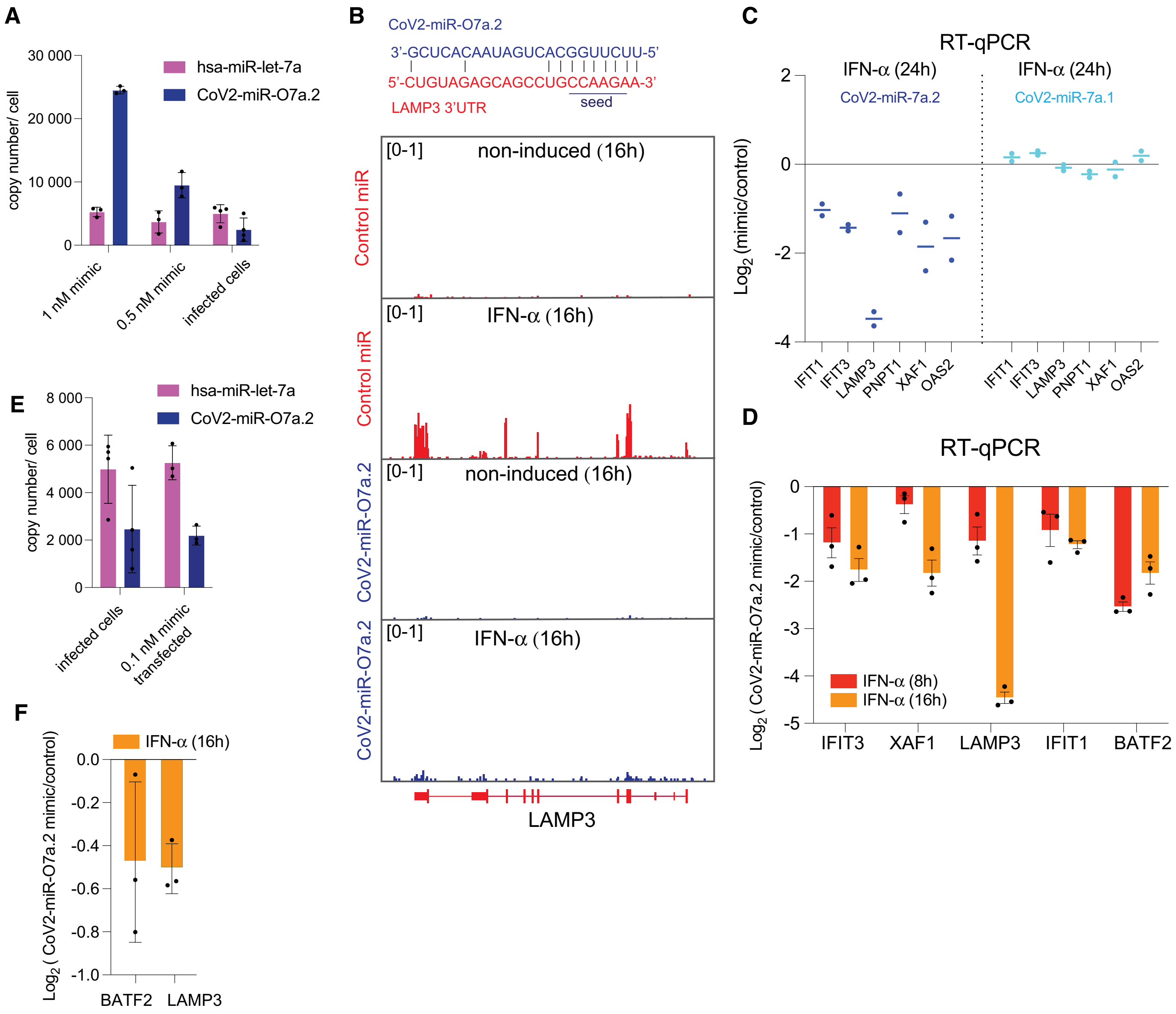

Figure EV5. Regulation of ISGs by SARS-CoV-2 miR-O7a across different time points of IFN-α treatment.

A Copy number per cell of CoV2-miR-O7a.2 transfected in A549-ACE2 cells for luciferase reporter experiment (Fig 4B) and hsa-let7a compared to SARS-CoV-2 infected cells. The mean and standard deviation of three experiments are shown. Data for virus-infected cells are shown again in this plot from Fig 3D for ease of comparison.

B Genomic view of the human LAMP3 gene showing normalized RNA-seq reads from non-induced and IFN-α-induced (for 16 h) A549-ACE2 cells transfected with CoV2-miR-O7a.2 or control mimics. The base pairing of CoV2-miR-O7a.2 to complementary 3′UTR site of LAMP3 is shown above and the seed region required for binding of miRNAs with the target is underlined.

C Log_2 fold change of expression of selected ISGs measured by RT-qPCR in IFN-α -treated A549-ACE2 cells transfected with CoV2-miR-O7a.2 or COV2-miR-O7a.1 compared to control mimic at 24 h upon IFN-α treatment. Line represents the average, and individual dots represent data from two independent experiments.

D Log_2 fold change of expression of selected ISGs measured by RT-qPCR in IFN-α -treated A549-ACE2 cells transfected with CoV2-miR-O7a.2 mimic compared to control mimic at 8 and 16 h upon IFN-α treatment. The mean and standard deviation of 3 experiments are shown.

E Copy number per cell of CoV2-miR-O7a.2 (0.1 nM) transfected in A549-ACE2 cells for 24 h along with hsa-let7a. Cells were then further induced by IFN-α for 16 h. The mean and standard deviation of three experiments are shown. Data for virus-infected cells are shown again in this plot from Fig 3D for ease of comparison.

F Log_2 fold change of expression of CoV2-miR-O7a targets, BATF2 and LAMP3, measured by RT-qPCR in IFN-α -treated A549-ACE2 cells transfected with CoV2-miR-O7a.2 (samples from Fig EV5E) compared to control mimic at 16 h upon IFN-α treatment. The mean and standard deviation of three experiments are shown.

Source data are available online for this figure.

 © 2021 The Authors
