# Supplementary material for: A virus‐derived microRNA targets immune response genes during SARS‐CoV‐2 infection
Source: EMBO Rep. 2021 Dec 16;23(2):e54341. doi: 10.15252/embr.202154341 (PMC8811647; doi:10.15252/embr.202154341)
Supplement: Supplementary file 15 [file EMBR-23-e54341-s009.pdf]

A virus-derived microRNA targets immune response genes during SARS-CoV-2 infection

Meetali Singh, Maxime Chazal, Piergiuseppe Quarato, Loan Bourdon, Christophe Malabat, Thomas Vallet, Marco Vignuzzi, Sylvie van der Werf, Sylvie Behillil, Flora Donati, Nathalie Sauvonnet, Giulia Nigro, Maryline Bourgine, Nolwenn Jouvenet, and Germano Cecere
DOI: 10.15252/embr.202154341

Corresponding author(s): Germano Cecere (germano.cecere@pasteur.fr) , Nolwenn Jouvenet (nolwenn.jouvenet@pasteur.fr)

Review Timeline:

Submission Date:	15th Nov 21
Editorial Decision:	19th Nov 21
Revision Received:	21st Nov 21
Accepted:	6th Dec 21

Editor: Martina Rembold

Transaction Report: This manuscript was transferred to EMBO reports following peer review at The EMBO Journal.

(Note: With the exception of the correction of typographical or spelling errors that could be a source of ambiguity, letters and reports are not edited. Depending on transfer agreements, referee reports obtained elsewhere may or may not be included in this compilation. Referee reports are anonymous unless the Referee chooses to sign their reports.)

1st Editorial Decision **19th Nov 2021**

Dear Dr. Cecere

Thank you for the transfer of your manuscript to EMBO Reports. Your study was reviewed and revised for The EMBO Journal. You provide evidence that SARS-CoV-2 encodes for two microRNAs that target interferon stimulated genes. We note that referee 1 remains concerned that the functional significance of miR-O7a.2 for SARS-CoV-2 infection and replication remains unclear at this stage. Furthermore, the referee indicated that no Northern blot was provided showing the mature viral miRNA and possibly a precursor stem-loop. We agree that the functional relevance of the miRNA remains to be determined and therefore all claims regarding the functional relevance for SARS-CoV-2 infection and the host's immune response must be significantly toned down, also in the title and abstract of the manuscript. Referee 1 concludes that SARS-CoV-2 appears to produce a small RNA that is Dicer dependent and the results should be described keeping this in mind and keeping speculations on the pathophysiological relevance for the discussion section.

We have also sought further expert advice on your manuscript regarding the evidence you provide that SARS-CoV-2 encodes and produces a miRNA. The advisor has expertise in miRNA biogenesis and function and concluded "[...] that the sequence data validation of the miRNA meet normal criteria." The advisor considered a Northern nice but not essential. I have copied the full comment below my signature for your information.

The advisor suggested to provide additional evidence whether the hairpin structure is conserved in the viral genomes (SARS-CoV-2 isoforms) and we ask you to provide this analysis in the revised manuscript. The advisor noted:

"The one additional evidence that I would like to see is that the hairpin structure is conserved in the viral genomes - where there is sequence variation in this structure their model predicts that there should be compensating changes on the opposite strand of the stem loop - or that the conservation of delta G is as great in this stem loop as in other structured regions of the genome.
If I understood correctly the miRNA precursor structure is specific to strains of SARS-CoV-2 but not to other coronaviruses. If the hairpin and its potential to derive miRNAs confers a fitness advantage (due to suppression of interferon responses) the structure should be conserved in the different isolates - the stability should be the same in the different isolates and any sequence polymorphism should be matched on both sides of the hairpin more often than expected by chance."

Taken together, we invite you to revise your manuscript for publication in EMBO reports. Please address all referee and advisor concerns in a complete point-by-point response.

There are also a number of points from the editorial side that need your attention:

1) Please provide up to 5 keywords

2) EV tables: Please provide the different supplementary tables as individual files.
- Table EV1, EV2, EV3, and EV5 should be uploaded as Dataset EV#. The legend remains part of the .xls file. The nomenclature is Dataset EV#.
- Table EV4 should be uploaded as Table EV1.
- The EV table legends should be removed from the Manuscript file.

3) EV figures: Please note that we can only typeset up to 5 EV figures. You can either combine two figures to reduce the total number to 5 or move some of the figures to an Appendix. You can of course also move all EV figures to an Appendix.

- For EV figures, the figure legends are part of the main manuscript text in a separate paragraph called "Expanded View Figure legends", just after the main "Figure legends" (please add paragraph titles).
All EV figures need to be supplied as individual, production quality files.

- The Appendix is a single PDF file called *Appendix*, which should start with a short Table of Content incl. page numbers. It contains the figures and their legends. Appendix figures should be referred to in the main text as: "Appendix Figure S1, Appendix Figure S2" etc. See detailed instructions regarding expanded view here:
<https://www.embopress.org/page/journal/14693178/authorguide#expandedview>

4) Please follow the model below for the Data availability section and please move it to the end of the Materials and Methods section. We also need a link that resolves to the dataset:

The accession numbers and database should be listed in a formal "Data Availability " section (placed after Materials & Method) that follows the model below (see also < https://www.embopress.org/page/journal/14693178/authorguide#dataavailability>). Please note that the Data Availability Section is restricted to new primary data that are part of this study.

Data availability

The datasets (and computer code) produced in this study are available in the following databases:

- RNA-Seq data: Gene Expression Omnibus GSE46843 (https://www.ncbi.nlm.nih.gov/geo/query/acc.cgi?acc=GSE46843)
- [data type]: [name of the resource] [accession number/identifier/doi] ([URL or identifiers.org/DATABASE:ACCESSION])

*** Note - All links should resolve to a page where the data can be accessed. ***

5) Please add a callout Fig EV6C.

6) References to preprints need to be clearly marked following this template:
• In-text citation: (preprint: NAME1 et al, YEAR)
• Author NAME1, Author NAME2, (YEAR) article title. bioRxiv doi: 1234/002.dfj123 [PREPRINT]

7) I attach to this email a related manuscript file with comments by our data editors. Please address all comments and upload a revised file with tracked changes with your final manuscript submission.

8) Moreover, we have noticed several quantification graphs based on duplicate measurements (n = 2) with given error bars and statistical comparisons, which is not good practice and against journal style.

9) Finally, EMBO reports papers are accompanied online by A) a short (1-2 sentences) summary of the findings and their significance, B) 2-3 bullet points highlighting key results and C) a synopsis image that is 550x200-600 pixels large (width x height) in .png format. You can either show a model or key data in the synopsis image. Please note that the size is rather small and that text needs to be readable at the final size. Please send us this information along with the revised manuscript.

We look forward to seeing a final version of your manuscript as soon as possible. Please use this link to submit your revision: https://embor.msubmit.net/cgi-bin/main.plex

With kind regards,

Martina Rembold, PhD
Senior Editor
EMBO reports

****

Comments from the advisor:

"[...] reviewer 1 is correct that there is no evidence that the miRNA has a role in the viral life cycle but that is not what the authors claim (at least if 'life cycle' means replication and turnover of viral RNA). They produce evidence that a small proportion of the viral genome is processed into the two miRNAs and that these miRNAs accumulate at a similar level to host encoded miRNAs. Correspondingly they use target mimic RNAs to provide evidence that the ISG mRNA targets of the miRNAs are indeed down regulated in infected cells.

I thought that the sequence data validation of the miRNA meet normal criteria - a northern would be nice but it is not essential.

There can be four reasons for miRNA or siRNA production from viral genomes - a role in the replication cycle, as part of antiviral defense by the host if the viral genome is targeted so that viral RNA accumulation is reduced, to target host (defense) genes or noise. Any of the first three is interesting and the authors of this paper have good supporting evidence of the third of these and I thought that the discussion was appropriately balanced.

The one additional evidence that I would like to see is that the hairpin structure is conserved in the viral genomes - where there is sequence variation in this structure their model predicts that there should be compensating changes on the opposite strand of the stem loop - or that the conservation of delta G is as great in this stem loop as in other structured regions of the genome.
If I understood correctly the miRNA precursor structure is specific to strains of SARS-CoV-2 but not to other coronaviruses. If the hairpin and its potential to derive miRNAs confers a fitness advantage (due to suppression of interferon responses) the structure should be conserved in the different isolates - the stability should be the same in the different isolates and any sequence polymorphism should be matched on both sides of the hairpin more often than expected by chance."

Referee #1:

This manuscript is only modestly improved over the first version and still fails to provide any evidence of physiological relevance for this small viral RNA.
To keep this short, I will focus on a couple of specific points:
1) As the authors note, SARS2 very effectively blocks the translation of host cell mRNAs shortly after infection (Finkel et al., 2021) which begs the question of what benefit the virus would derive from a miRNA that only accumulates later in infection and that only inhibits target mRNA expression by a rather modest 2-fold (figure 4B). So we REALLY need to see the authors test a SARS2 mutant bearing a mutant form of this putative viral miRNA that is either no longer able to target any ISGs or that is disrupted for stem formation. A clean virus phenotype and/or an increase in ISG expression in mutant virus infected cells, relative to WT is predicted and needs to be shown.
2) Dicer will cleave ANY dsRNA stem, so the new data in figure 3B are meaningless and should be deleted. The question is, how does Dicer access this stem when it flanked by ssRNA sequences in the viral genome? I notice that no Northern blot has been presented showing the mature viral miRNA (which should be easy if it is expressed at the level proposed) and this Northern might also reveal some form of precursor pre-miRNA stem-loop. At present, these data are reminiscent of the putative miRNA rerived from the HIV_1 TAR element, which proved to be basically an artifact.
In conclusion, yes SARS2 produces a small RNA that is Dicer dependent but my guess is this plays no role whatever in the viral life cycle. This issue needs to be effectively addressed prior to publication.

Author's response to Reviewers, 1st revision

Referee #1:

In this manuscript, Singh et al first present small RNA seq data from SARS2 infected cells and report the presence of reads from a region within ORF7. These candidate miRNAs, termed miR-7a-1 and -2, are somewhat conserved and derive from a potential pre-miRNA-like stem loop structure. An artificial mimic of miR-7a-2 was shown to affect the interferon response, possibly by targeting BATF2. Despite what is claimed on page 4, small RNA-seq has been performed in cells infected by a wide range of RNA viruses, including picorna, orthomyxo, paramyxo, rhabdo and flavi viruses without any viral miRNAs being discovered. So, evidence that SARS2 encodes a miRNA would be of great interest. However, this manuscript falls far short of proving this point and is most consistent with the idea that these small RNAs derive from Dicer cleavage of a short viral RNA stem-loop that remains stable, due to it's secondary structure, after exonucleolytic degradation of the SARS2 RNA genome. This kind of thing has been seen before, eg. with the HIV-1 TAR element, yet none of these miRNA-like small RNAs have been shown to have any physiological relevance. I have the following specific concerns:

We would like to thank Referee #1 for the insightful review and for helping us to strengthen the conclusions of our manuscript. We have addressed the specific referees' concerns below.

1) A key characteristic of real miRNAs is that they have precise ends while miRNA-like small RNAs that derive from RNA degradation products have ragged ends. So we need to see the precise structure of all the miR-7a-1 and -2 reads in figure 1, not just a representative read.

We thank Referee #1 for this comment that helps us to highlight our finding on the viral miRNA. In figure EV1F-G, and EV2G-H, we have analyzed the length of the CoV2-miRNA-7a.2 reads at their 5'or 3' end. The results show that the majority of CoV2-miRNA-7a.2 reads have precise end, which is a characteristic of bonafide miRNA.

2) Most miRNAs require cleavage by Drosha to generate the pre-miRNA cleaved by Dicer and this requires an ~33bp stem. The RNA structures shown in Fig. 2A are too short to be substrates cleaved by Drosha and Dicer is an exonuclease, NOT an endonuclease. So there is no way for Dicer to cleave the stem-loops shown here unless the flanking ssRNA sequences had been nibbled away by cellular exonucleases. If the authors really believe that this stem-loop is a direct substrate for miRNA biogenesis, they should express this region of the SARS2 genome using an expression vector and show that functional miRNAs are generated.

We thank Referee #1 for this comment that helps us to clarify the role of Dicer in processing the RNA structure shown in Fig. 2A. In our manuscript, we have shown that DICER knockdown leads to reduced levels of the SARS-CoV-2 miR-7a1 and 7a2 at a similar level as that of endogenous miRNA let7a. This result led us to propose that Dicer can process the structure shown in Fig. 2A. To address Referee #1 concern that the RNA structure shown in Fig. 2A is a substrate cleaved by Dicer, we have performed in vitro assay using the stem-loop precursor structure and purified human Dicer enzyme. The new results in Fig. 3B show that the precursor can generate the viral miRNA (detected by stem-loop RT-qPCR) in a time-course experiment with human Dicer. In contrast, the same

miRNA sequence embedded in a mutated precursor that does not form a stem-loop structure fails to be processed by Dicer. In addition to our results, a manuscript deposited on Biorxiv by Joan Steitz lab, which has a very similar finding, shows that the transfection of a 100nt precursor generates the same viral miRNA in a drosha-independent manner. Together with the results by the Steitz lab, our results suggest that the viral miRNA is produced in a drosha-independent (Steiz study) but dicer-dependent (our study) manner.

There are many examples of drosha-independent miRNA biogenesis discovered in many organisms ranging from plants to humans, which still require the endoribonuclease Dicer. Many of these miRNAs are processed from introns (mirtrons), and others can even be generated from snoRNA and tRNA fragments that form a stem-loop structure recognized by Dicer (among the many reviews on the topic, more information can be found in Treiber et al., Nature Reviews Molecular Cell Biology 2018). In these cases of drosha-independent miRNA biogenesis, cellular exonucleases (such as the exosome) can trim the 3' end and the 5' end of the putative miRNA precursor. We believe that the miRNA generated from the SARS-CoV-2 RNA genome can similarly result from a drosha-independent miRNA biogenesis process.

3) The effect of Dicer knock down on miR-7a-1 and -2 biogenesis is very modest. Dicer KO cell lines have been published, and with the advent of CRISPR/Cas, it would be trivial to generate a Dicer KO in a SARS2-permissive cell line to see if ALL miR-7a reads are lost.

We agree with Referee #1 that an experiment with Dicer KO would be nice to complement our Dicer knockdown experiments. Unfortunately, the only Human Dicer KO cell line available (Bogerd et al, RNA, 2014) are HEK-293T cells, which are not competent for SARS-CoV-2 viral infection. We tried to express the ACE2 receptor in those cells by transient transfection prior to infect them with SARS-CoV-2. Unfortunately, these cells are fragile and the efficiency of infection was too low to allow us to measure the viral miRNAs. We have also tried to generate CRISPR/Cas9 Dicer KO in A549-ACE2 cells. We have requested those cell lines from a Company specialized in creating human KO cell lines. However, even if they were able to generate cells with edited Dicer alleles, during the propagation of this edited cell pools most of them had WT expression of Dicer. It is possible that Dicer KO cells are less fit than WT cells, resulting in a severe reduction in the KO cells in the pool overtime. Nonetheless, we hope that the referee #1 appreciate that even if DICER knockdown by siRNAs is modest, the level of reduction of the SARS-CoV-2 miR-7a1 and 7a2 is similar to the endogenous and abundant human miRNA let7a. This results together with the experiment on dicer in vitro processing discussed in point 2 should convince the referee#1 that Dicer can promote the accumulation of the viral miRNA.

4) It is well established that miRNAs act stoichiometrically, so expression levels >200/cell, at minimum, are required for activity. I find the data in figure 4, which putatively addresses this issue, difficult to understand. Instead, assuming there are 50,000 miRNAs per cell, the authors can estimate the copy number of these putative viral miRNAs per cell using their deep sequencing data.

We thank Referee #1 for helping us in clarifying our results shown in Figure 4. We have now used a standard method to quantify miRNA copy number per cell with a spike-in synthetic small RNAs standard curve (Chen et al. Nucleic Acids Research, 2005). Our results (fig. 3D) show that we have approximately 2000 copies of viral miRNA per cell, which is ten times higher than the minimum of 200/cell required for activity as mentioned by Referee #1. It is also important to note that the viral miRNA copy number is in the range of the let-7 human miRNA, which is among the most abundant miRNA in the cell. Given that the viral miRNA is loaded into AGOs with similar efficiency, we believe it might have a biological function as many other highly abundant endogenous human miRNAs.

5) The use of massively overexpressed miRNA mimics in figures 5 and 6 is totally inadequate to prove that the same cellular mRNAs are downregulated by physiological levels of the putative viral miRNAs produced in infected cells. I suggest that the authors generate a mutant SARS2 that bears a couple of silent mutations that do not affect any underlying ORF but mutate the miRNA seed sequences. They can then infect cells and show that the mutant does not affect the expression of their proposed cellular mRNA targets while the WT virus does. If this is too much to ask, then they should transfect SARS2-infected cells with indicator constructs in which a luciferase gene has a 3'UTR derived from BATF2 in which the proposed target for miR-7a-1 is either present or mutated. Only in this way can we see that physiological levels of this small RNA are functionally relevant (which I do not believe is the case).
In conclusion, this paper falls far short of proving it's point and is not publishable in it's current form.

We understand the referees' concern about using overexpressed miRNA mimics utilized in many studies on miRNA function. We have now used a concentration of miRNA mimic that is similar to the viral miRNA (~2000 copies/cells). In these conditions, BATF2 and LAMP3 expression were downregulated, two of the viral miRNA targets with extensive complementarity (fig. EV5E-F).

We have also tried to perform experiments using luciferase reporter assay and viral infection, as suggested by Referee #1. Unfortunately, the viral infection per se is causing a global shut-off of luciferase and renilla expression, which prevented us to test the role of viral miRNA in this assay. This global shut-off issue with renilla luciferase reporter has also been mentioned by the Steitz lab in their Biorxiv manuscript. To overcome this issue, we have used the transfection of the miRNA mimic with the different concentrations (fig 4B) and showed that the miRNA mimic can significantly downregulate the luciferase in a construct with a fully complementary site compared to the one mutated in the seed region (the first 8nt).

Referee #2:

The authors of the manuscript „A virus-encoded microRNA contributes to evade innate immune response during SARS-CoV-2 infection" provide evidence for the first time that the genome of the RNA virus SARS Coronavirus 2 (SARS-CoV-2) encodes a micro RNA (miRNA) that is processed by the host miRNA pathway and influences gene expression of the human host.

The authors first identify two 22 nucleotide long small RNAs derived from open reading frame ORF7a in the SARS-CoV-2 genome in intestinal-derived and pulmonary-derived human cell lines and show that they are two isoforms of the same virus-encoded miRNA, and designated them CoV2-miR-7a.1 and CoV2-miR-7a.2. Interestingly, these miRNAs are highly conserved between different SARS-CoV-2 variants and also conserved in the genome of Coronaviruses from Pangolin and Bats, but not in that of SARS-CoV. Using an siRNA approach the authors can demonstrate that human Dicer is involved in SARS-CoV-2 miRNA processing. Next, they identified the 3'-UTR region of the cellular transcription factor BATF2, which is an interferon-stimulated gene (ISG), as a potential target of CoV2-miR-7 due to high sequence homology and show experimentally that mRNA levels of the ISGs BATF2 and LAMP3 are significantly downregulated upon transfection of CoV2-miR-7a.2, but not CoV2-miR-7a.1, after IFNalpha stimulation. By RNA-seq they demonstrate that multiple ISGs are affected by CoV2-miR-7. Lastly, the authors are able to detect CoV2-miR-7 in 2D human colon organoids and in nasopharyngeal swabs of COVID-19 patients. Overall, this is a nice study, it is written well and the figures, legends and M+M section are well presented. However, I noticed some shortcomings that are summarized below.

We would like to thank Referee #2 for appreciating the quality of our study and the presentation of our findings. We also would like to thank Referee #2 for helping us improve the manuscript text.

Major comments:

The discussion is extremely short, and the result section describing figures 5 and 6 is also written in a minimalist way. I was wondering why the authors first claim that they identified the 3'-UTR of BATF2 as possible CoV2-miR-7 target, but then identify multiple ISGs as targets by RNA-seq, without really explaining/commenting/discussing this finding.

As suggested by Referee #2, we have expanded the results and discussion sections.

Apart from ISGs, were also other genes identified that were differentially regulated by CoV2-miR-7?

We have analyzed gene expression changes upon transfection of SARS-CoV-2 miR-7a1, and 7a2 mimics upon interferon stimulation to mimic viral infection. We have observed the maximum effect of SARS-CoV-2 miR-7a1 and 7a2 mimics on suppressing ISGs in a sequence-dependent manner. We cannot exclude that the viral miRNAs can potentially target other non ISGs genes. Still, we have not found a specific signature for that, and we have focused on ISGs given the relevance of these genes for the progression of COVID-19 disease. We have provided in a supplemental table the gene expression changes for all the human genes.

The authors only focus on ISGs without commenting on other host genes. This leaves me with the question: does CoV2-miR-7 directly target ISGs, or does it target a cellular gene whose lower expression can explain lower ISG expression?

We have computationally determined target sites for SARS-CoV-2 miR-7a1 and 7a2. We can confirm that the ISGs contain the target sites in their 3’UTR and the level of suppression depends on the target site sequence conservation (8mer vs. 6mer, for instance). We have clarified the targeting rules of miRNAs on ISGs in our results and discussion sections.

An experiment showing that the SARS-CoV-2 miRNA really binds to a host sequence would shed light on this question.

We have now shown using luciferase reporters that the downregulation of target sequences depends on the seed region, similar to what we have observed on ISGs (Figure 4A-B).

Do all ISGs that were found to be regulated show a certain degree of sequence homology to CoV2-miR-7? Do the ISGs that are regulated have something in common - the 3'-UTR sequence or the promoter/enhancer sequence?

We regret that this was not clear in the result section. In fact, our results (Fig 5f) show that many of the ISGs have target site for SARS-CoV-2 miR-7a2 in their 3’UTR. Not all the ISGs show 100 % conservation of the miRNA target sequence and we see the degree of suppression of their expression by SARS-CoV-2 miR-7a2 correlates with the conservation of miRNA target sequence in the 3’UTR.

In the discussion the authors should discuss their work in the light of the recent literature, e.g. elaborating on other viral miRNAs and their targets, are other viruses targeting host genes via their 3'-UTR etc. (in other word: the discussion should be improved). Literature on computer predictions for SARS-CoV-2 miRNAs exists and maybe those papers could be discussed as well.

We have included the suggestions of the reviewer to modify the discussion.

Minor comments:

1. Can you please explain better the nature of the 8mer, 7merm8, 7mer-A1 (see Figure 5F), maybe by showing their sequence.

We have included a schematic and details of miRNA binding sequence conservation.

2. Figure 3: I would suggest to fuse it with Figure 2 - it does not really stand on its own and is tightly linked to Figure 2.

Following Referee #2 suggestion we have now moved the Figure 3 in supplementary figures.

3. Figure 6: The figure is difficult to digest - the gene label in A and B is identical and it takes a while to understand the color scheme - hence, wouldn't it make sense to put A

(intensity 10 and 5) and B (intensity 2 and -2) in one figure? It then becomes easier to understand.

The scales and comparison for two panels are different and will therefore be not right to put in one figure. However, keeping in view, referee suggestion we have labeled the figure better to avoid any confusion.

4. Figure 4C: In the text it says that human and SARS-CoV2 miRNA levels are identical - I would suggest to tone that down slightly, since levels are not really identical.

We have modified the text keeping in view reviewer's suggestions.

Comments from the advisor:

"[...] reviewer 1 is correct that there is no evidence that the miRNA has a role in the viral life cycle but that is not what the authors claim (at least if 'life cycle' means replication and turnover of viral RNA). They produce evidence that a small proportion of the viral genome is processed into the two miRNAs and that these miRNAs accumulate at a similar level to host encoded miRNAs. Correspondingly they use target mimic RNAs to provide evidence that the ISG mRNA targets of the miRNAs are indeed down regulated in infected cells.

I thought that the sequence data validation of the miRNA meet normal criteria - a northern would be nice but it is not essential.

There can be four reasons for miRNA or siRNA production from viral genomes - a role in the replication cycle, as part of antiviral defense by the host if the viral genome is targeted so that viral RNA accumulation is reduced, to target host (defense) genes or noise. Any of the first three is interesting and the authors of this paper have good supporting evidence of the third of these and I thought that the discussion was appropriately balanced.

We would like to thank the Advisor for the positive feedback on our manuscript and for appreciating our results and discussion.

The one additional evidence that I would like to see is that the hairpin structure is conserved in the viral genomes - where there is sequence variation in this structure their model predicts that there should be compensating changes on the opposite strand of the stem loop - or that the conservation of delta G is as great in this stem loop as in other structured regions of the genome.

If I understood correctly the miRNA precursor structure is specific to strains of SARS-CoV-2 but not to other coronaviruses. If the hairpin and its potential to derive miRNAs confers a fitness advantage (due to suppression of interferon responses) the structure should be conserved in the different isolates - the stability should be the same in the different isolates and any sequence polymorphism should be matched on both sides of the hairpin more often than expected by chance."

We thank the Advisor for this suggestion. We have now included the analysis suggested by the Advisor in the new Fig. EV3. We have compared the sequences of the first 70 nt of ORF7a, which are involved in stem-loop formation. We see that in the representative sequences of various SARS-CoV-2 variants, the entire 70 nt sequences show 100 % conservation, except for four variants showing single point mutations at different positions. We analyzed the predicted structures and their minimum free energy. We found that observed mutations in these four variants do not impact either the stem-loop formation or the minimum free energy of the structure.

Additionally, we had performed sequence conservation analysis using about 4 million available sequences of the various SARS-CoV-2 variants and found that the 1st 70 nucleotides of ORF7a show a higher percentage of conservation than the rest of the ORF7a sequence (Fig. 2C and Appendix Fig.1).

Referee #1:

This manuscript is only modestly improved over the first version and still fails to provide any evidence of physiological relevance for this small viral RNA.
To keep this short, I will focus on a couple of specific points:
1) As the authors note, SARS2 very effectively blocks the translation of host cell mRNAs shortly after infection (Finkel et al., 2021) which begs the question of what benefit the virus would derive from a miRNA that only accumulates later in infection and that only inhibits target mRNA expression by a rather modest 2-fold (figure 4B). So we REALLY need to see the authors test a SARS2 mutant bearing a mutant form of this putative viral miRNA that is either no longer able to target any ISGs or that is disrupted for stem formation. A clean virus phenotype and/or an increase in ISG expression in mutant virus infected cells, relative to WT is predicted and needs to be shown.

We would like to argue that SARS-CoV-2 uses a multipronged approach for suppressing host response resulting in a multi-organ pathology, making the virus so successful. Given that miRNAs act to fine-tune and not suppress the expression of their target, this level of downregulation is expected. It has also been proven in many previous reports that such a level of downregulation is sufficient to cause critical phenotypic consequences. Moreover, we cannot predict the level of downregulation of such miRNA targets in the actual tissue or cellular context of action during human viral infection.

Besides the fact that it is not easy to obtain authorization to manipulate the SARS-CoV-2 genome, the size of SARS-CoV-2 (almost 30 Kb) renders such manipulation very challenging. Indeed, very few laboratories worldwide manage to create a mutated version of the virus. Furthermore, it is also not trivial to choose a good cellular model for testing the role of WT and mutated viruses in suppressing immune responsive genes. For these reasons, we decided to provide an alternative experiment proposed by Reviewer #1 using a luciferase reporter with mutated or WT miRNA binding sites.

2) Dicer will cleave ANY dsRNA stem, so the new data in figure 3B are meaningless and should be deleted. The question is, how does Dicer access this stem when it flanked by ssRNA sequences in the viral genome? I notice that no Northern blot has been presented showing the mature viral miRNA (which should be easy if it is expressed at the level proposed) and this Northern might also reveal some form of precursor pre-miRNA stem-loop. At present, these data are reminiscent of the putative miRNA rerived from the HIV_1 TAR element, which proved to be basically an artifact.

In conclusion, yes SARS2 produces a small RNA that is Dicer dependent but my guess is this plays no role whatever in the viral life cycle. This issue needs to be effectively addressed prior to publication.

In the initial review, Reviewer #1 claimed that *"Most miRNAs require cleavage by Drosha to generate the pre-miRNA cleaved by Dicer, and this requires an ~33bp stem. The RNA structures shown in Fig. 2A are too short to be substrates cleaved by Drosha, and Dicer is an exonuclease, NOT an endonuclease. So there is no way for Dicer to cleave the stem-loops shown here unless*

the flanking ssRNA sequences had been nibbled away by cellular exonucleases." Despite Reviewer #1 opinion that Dicer is an exonuclease, decades of research have shown that Dicer is an endonuclease. To show that Dicer can indeed cleave the predicted stem-loop structure shown in our manuscript, we demonstrated in our revised manuscript that Dicer cleaves such structure by an established in vitro cleavage assay. These results are not "meaningless" because they helped conclude that "SARS2 produces a small RNA that is Dicer dependent". In our revised manuscript, we have discussed a possible Drosha-independent and Dicer-dependent miRNA biogenesis mechanism and how cellular nucleases can generate a precursor that is then cleaved by Dicer. We think that identifying such cellular nucleases that act upstream of Dicer in generating SARS-CoV-2 miRNA is out of the scope of the present study and will be addressed in future works. It is worth mentioning that despite many reported cases of endogenous drosha-independent and dicer-dependent miRNAs, the biogenesis of such miRNAs is still not fully understood.

Regarding the request for a northern blot experiment, we agree with the Advisor that this is not a crucial experiment. Moreover, we have currently disposed of the use of radioactivity in our lab to align our research with more environmental-friendly experiments, which can give similar results (such as small RNA sequencing and RT-qPCR used in our manuscript).
Therefore, requesting such authorization again will take months. Moreover, a complementary study by the Steitz lab deposited on Biorxiv has already shown such northern blotting experiments.

Dr. Germano Cecere
Pasteur Institute
25 Rue du Docteur roux
Paris, Ile de france 75724
France

Dear Germano,

As you know, we have meanwhile heard back from former referee 2, who concluded that you have adequately addressed his/her concerns that were raised during the first round of peer review. Thank you also for updating the methods sections. I am now very pleased to accept your manuscript for publication in the next available issue of EMBO reports. Thank you for your contribution to our journal.

At the end of this email I include important information about how to proceed. Please ensure that you take the time to read the information and complete and return the necessary forms to allow us to publish your manuscript as quickly as possible.

As part of the EMBO publication's Transparent Editorial Process, EMBO reports publishes online a Review Process File to accompany accepted manuscripts. As you are aware, this File will be published in conjunction with your paper and will include the referee reports, your point-by-point response and all pertinent correspondence relating to the manuscript.

If you do NOT want this File to be published, please inform the editorial office within 2 days, if you have not done so already, otherwise the File will be published by default [contact: emboreports@embo.org]. If you do opt out, the Review Process File link will point to the following statement: "No Review Process File is available with this article, as the authors have chosen not to make the review process public in this case." Please note that the author checklist will still be published even if you opt out of the transparent process.

Thank you again for your contribution to EMBO reports and congratulations on a successful publication. Please consider us again in the future for your most exciting work.

Kind regards,

Martina

Martina Rembold, PhD
Senior Editor
EMBO reports

****

THINGS TO DO NOW:

You will receive proofs by e-mail approximately 2-3 weeks after all relevant files have been sent to our Production Office; you should return your corrections within 2 days of receiving the proofs.

Please inform us if there is likely to be any difficulty in reaching you at the above address at that time. Failure to meet our deadlines may result in a delay of publication, or publication without your corrections.

All further communications concerning your paper should quote reference number EMBOR-2021-54341V2 and be addressed to emboreports@wiley.com.

Should you be planning a Press Release on your article, please get in contact with emboreports@wiley.com as early as possible, in order to coordinate publication and release dates.

EMBO PRESS

YOU MUST COMPLETE ALL CELLS WITH A PINK BACKGROUND

PLEASE NOTE THAT THIS CHECKLIST WILL BE PUBLISHED ALONGSIDE YOUR PAPER

Corresponding Author Name: Germano Cecere
Journal Submitted to: EMBO Reports
Manuscript Number: EMBOR-2021-54341V2

Reporting Checklist For Life Sciences Articles (Rev. June 2017)

This checklist is used to ensure good reporting standards and to improve the reproducibility of published results. These guidelines are consistent with the Principles and Guidelines for Reporting Preclinical Research issued by the NIH in 2014. Please follow the journal's authorship guidelines in preparing your manuscript.

A- Figures
1. Data
The data shown in figures should satisfy the following conditions:

the data were obtained and processed according to the field's best practice and are presented to reflect the results of the experiments in an accurate and unbiased manner.
figure panels include only data points, measurements or observations that can be compared to each other in a scientifically meaningful way.
graphs include clearly labeled error bars for independent experiments and sample sizes. Unless justified, error bars should not be shown for technical replicates.
if n< 5, the individual data points from each experiment should be plotted and any statistical test employed should be justified
Source Data should be included to report the data underlying graphs. Please follow the guidelines set out in the author ship guidelines on Data Presentation.

2. Captions

Each figure caption should contain the following information, for each panel where they are relevant:

a specification of the experimental system investigated (eg cell line, species name).
the assay(s) and method(s) used to carry out the reported observations and measurements
an explicit mention of the biological and chemical entity(ies) that are being measured.
an explicit mention of the biological and chemical entity(ies) that are altered/varied/perturbed in a controlled manner.

the exact sample size (n) for each experimental group/condition, given as a number, not a range;
a description of the sample collection allowing the reader to understand whether the samples represent technical or biological replicates (including how many animals, litters, cultures, etc.).
a statement of how many times the experiment shown was independently replicated in the laboratory.
definitions of statistical methods and measures:

- common tests, such as t-test (please specify whether paired vs. unpaired), simple $\chi 2$ tests, Wilcoxon and Mann-Whitney tests, can be unambiguously identified by name only, but more complex techniques should be described in the methods section;
- are tests one-sided or two-sided?
- are there adjustments for multiple comparisons?
- exact statistical test results, e.g., P values = x but not P values < x;
- definition of 'center values' as median or average;
- definition of error bars as s.d. or s.e.m.

Any descriptions too long for the figure legend should be included in the methods section and/or with the source data.

In the pink boxes below, please ensure that the answers to the following questions are reported in the manuscript itself. Every question should be answered. If the question is not relevant to your research, please write NA (non applicable). We encourage you to include a specific subsection in the methods section for statistics, reagents, animal models and human subjects.

USEFUL LINKS FOR COMPLETING THIS FORM

http://www.antibodypedia.com
http://1degreebio.org
http://www.equator-network.org/reporting-guidelines/improving-bioscience-research-repor
http://grants.nih.gov/grants/olaw/olaw.htm
http://www.mrc.ac.uk/Ourresearch/Ethicsresearchguidance/Useofanimals/index.htm
http://ClinicalTrials.gov
http://www.consort-statement.org
http://www.consort-statement.org/checklists/view/32-consort/66-title
http://www.equator-network.org/reporting-guidelines/reporting-recommendations-for-tumo
http://datadryad.org
http://figshare.com
http://www.ncbi.nlm.nih.gov/gap
http://www.ebi.ac.uk/ega
http://biomodels.net/
http://biomodels.net/miriam/
http://jjj.biochem.sun.ac.za
https://osp.od.nih.gov/biosafety-biosecurity-and-emerging-biotechnology/
http://www.selectagents.gov/

B- Statistics and general methods

Please fill out these boxes (Do not worry if you cannot see all your text once you press return)

1.a. How was the sample size chosen to ensure adequate power to detect a pre-specified effect size?	For total sRNA-seq, RNA-seq experiments, qPCRs and IP atleast 100 000 cells were used. This sample size has been empirically evaluated to be sufficient to obtain large amount of RNAs or proteins content, sufficient for multiple applications. All the experiments shown in this study were performed independently at least two times and no inconsistent results were observed. All attempts at replication were successful.
1.b. For animal studies, include a statement about sample size estimate even if no statistical methods were used.	not applicable
2. Describe inclusion/exclusion criteria if samples or animals were excluded from the analysis. Were the criteria pre-established?	not applicable
3. Were any steps taken to minimize the effects of subjective bias when allocating animals/samples to treatment (e.g. randomization procedure)? If yes, please describe.	Our experiments were not randomized and controlling covariates was not necessary. For all experiments, control vs. experimental samples were treated in parallel. Samples were allocated into different groups based on treatment condition or infection status of samples.
For animal studies, include a statement about randomization even if no randomization was used.	not applicable
4.a. Were any steps taken to minimize the effects of subjective bias during group allocation or/and when assessing results (e.g. blinding of the investigator)? If yes please describe.	For gene-expression change analysis sample identities were blinded.
4.b. For animal studies, include a statement about blinding even if no blinding was done	not applicable
5. For every figure, are statistical tests justified as appropriate?	We have chosen appropriate statistical test for each figure. Specific details of the tests such as parametric or non parametric, two tailed are mentioned in the respective figure legends.
Do the data meet the assumptions of the tests (e.g., normal distribution)? Describe any methods used to assess it.	Data were analysed using Prism software, and appropriate tests were performed in the course of analysis. Whenever the sample size is high, it was assumed to follow normal distribution.

Is there an estimate of variation within each group of data?	standard deviations of standard error are shown for the variation within each group of data in respective figures.
Is the variance similar between the groups that are being statistically compared?	yes, the variance was simialr between the groups that are being statistically compared.

C- Reagents

6. To show that antibodies were profiled for use in the system under study (assay and species), provide a citation, catalog number and/or clone number, supplementary information or reference to an antibody validation profile. e.g., Antibodypedia (see link list at top right), 1DegreeBio (see link list at top right).	We have included the catalog numbers for both the antibody used in method section.
7. Identify the source of cell lines and report if they were recently authenticated (e.g., by STR profiling) and tested for mycoplasma contamination.	We have indicated this in methods. Human lung A549-ACE2 cells, which have been modified to stably express ACE2 via lentiviral transduction, were generated in the laboratory of Pr. Olivier Schwartz, (Institut Pasteur, Paris, France). Human colorectal adenocarcinoma Caco-2 and African green monkey Vero E6 cells were purchased from ATCC.

* for all hyperlinks, please see the table at the top right of the document

D- Animal Models

8. Report species, strain, gender, age of animals and genetic modification status where applicable. Please detail housing and husbandry conditions and the source of animals.	not applicable
9. For experiments involving live vertebrates, include a statement of compliance with ethical regulations and identify the committee(s) approving the experiments.	not applicable
10. We recommend consulting the ARRIVE guidelines (see link list at top right) (PLoS Biol. 8(6), e1000412, 2010) to ensure that other relevant aspects of animal studies are adequately reported. See author guidelines, under 'Reporting Guidelines'. See also: NIH (see link list at top right) and MRC (see link list at top right) recommendations. Please confirm compliance.	not applicable

E- Human Subjects

11. Identify the committee(s) approving the study protocol.	National Reference Center for Respiratory Viruses (NRC) at Institut Pasteur (WHO reference laboratory providing confirmatory testing for COVID-19).
12. Include a statement confirming that informed consent was obtained from all subjects and that the experiments conformed to the principles set out in the WMA Declaration of Helsinki and the Department of Health and Human Services Belmont Report.	The investigations were carried out in accordance with the General Data Protection Regulation (Regulation (EU) 2016/679 and Directive 95/46/EC) and the French data protection law (Law 78–17 on 06/01/1978 and Décret 2019–536 on 29/05/2019).
13. For publication of patient photos, include a statement confirming that consent to publish was obtained.	not applicable
14. Report any restrictions on the availability (and/or on the use) of human data or samples.	not applicable
15. Report the clinical trial registration number (at ClinicalTrials.gov or equivalent), where applicable.	not applicable
16. For phase II and III randomized controlled trials, please refer to the CONSORT flow diagram (see link list at top right) and submit the CONSORT checklist (see link list at top right) with your submission. See author guidelines, under 'Reporting Guidelines'. Please confirm you have submitted this list.	not applicable
17. For tumor marker prognostic studies, we recommend that you follow the REMARK reporting guidelines (see link list at top right). See author guidelines, under 'Reporting Guidelines'. Please confirm you have followed these guidelines.	not applicable

F- Data Accessibility

18: Provide a "Data Availability" section at the end of the Materials & Methods, listing the accession codes for data generated in this study and deposited in a public database (e.g. RNA-Seq data: Gene Expression Omnibus GSE39462, Proteomics data: PRIDE PXD000208 etc.) Please refer to our author guidelines for 'Data Deposition'.<br><br>Data deposition in a public repository is mandatory for:<br>a. Protein, DNA and RNA sequences<br>b. Macromolecular structures<br>c. Crystallographic data for small molecules<br>d. Functional genomics data<br>e. Proteomics and molecular interactions	Data has been submitted on GEO and accession details have been provided in Data Availabilty.
19. Deposition is strongly recommended for any datasets that are central and integral to the study; please consider the journal's data policy. If no structured public repository exists for a given data type, we encourage the provision of datasets in the manuscript as a Supplementary Document (see author guidelines under 'Expanded View' or in unstructured repositories such as Dryad (see link list at top right) or Figshare (see link list at top right).	Data has been submitted on GEO and accession details have been provided in Data Availabilty.
20. Access to human clinical and genomic datasets should be provided with as few restrictions as possible while respecting ethical obligations to the patients and relevant medical and legal issues. If practically possible and compatible with the individual consent agreement used in the study, such data should be deposited in one of the major public access-controlled repositories such as dbGAP (see link list at top right) or EGA (see link list at top right).	sRNA-seq data from nasal swabs has been deposited on GEO.
21. Computational models that are central and integral to a study should be shared without restrictions and provided in a machine-readable form. The relevant accession numbers or links should be provided. When possible, standardized format (SBML, CellML) should be used instead of scripts (e.g. MATLAB). Authors are strongly encouraged to follow the MIRIAM guidelines (see link list at top right) and deposit their model in a public database such as Biomodels (see link list at top right) or JWS Online (see link list at top right). If computer source code is provided with the paper, it should be deposited in a public repository or included in supplementary information.	Data has been analysed using publically availble codes with citations in the methods section.

G- Dual use research of concern

22. Could your study fall under dual use research restrictions? Please check biosecurity documents (see link list at top right) and list of select agents and toxins (APHIS/CDC) (see link list at top right). According to our biosecurity guidelines, provide a statement only if it could.	not applicable
